# Signal-Corrected LC–MS/MS Approaches in Amino Acid and Biogenic Amine Profiling for Chemometric Characterization of Commercial Dark Chocolates According to Cocoa Content and Manufacturer

**DOI:** 10.3390/molecules31152597

**Published:** 2026-07-25

**Authors:** Laura V. Morales, Sonia Sentellas, Javier Saurina

**Affiliations:** 1 Department of Chemical Engineering and Analytical Chemistry, Universitat de Barcelona, Martí I Franquès 1-11, 08028 Barcelona, Spain; 2 Institut de Recerca en Nutrició i Seguretat Alimentària, Universitat de Barcelona, 08921 Santa Coloma de Gramenet, Spain; 3 Serra Húnter Fellow Programme, Generalitat de Catalunya, Via Laietana 2, 08003 Barcelona, Spain

**Keywords:** amino acids, biogenic amines, LC-MS/MS profiling, chocolate classification, data drift correction, chemometrics

## Abstract

The chemical composition of chocolate is influenced by multiple factors, including cocoa genotype, fermentation, roasting, and formulation, which complicate the identification of reliable compositional descriptors. In this study, amino acid and biogenic amine profiles were determined by LC–MS/MS in 91 commercial dark chocolates (56–100% cocoa content) to assess their potential for product characterization. Because the analytical sequence extended over several days, different signal-correction strategies (including internal standard normalization and quality-control-based drift correction) were evaluated to compensate for instrumental variability prior to chemometric analysis. Signal reductions of up to 91% were observed, and the selected correction procedures improved measurement precision by up to 18-fold. A total of 19 amino acids and 8 amines were detected, with tyrosine, hydroxyproline, proline, leucine, phenylalanine, and valine being the most abundant compounds. Principal Component Analysis revealed a compositional gradient primarily associated with cocoa content, whereas classifications based on cocoa variety, geographical origin, and certification status showed limited discrimination. In contrast, Partial Least Squares-Discriminant Analysis achieved a balanced classification accuracy of 75.2% under repeated M-fold cross-validation when classifying samples according to manufacturer. Furthermore, permutation tests were carried out, confirming that the observed classification performance was highly unlikely to arise by chance. These findings indicate that amino acid and biogenic amine profiles in commercial dark chocolates are influenced mainly by cocoa content and manufacturer-related factors, whereas the effects of cocoa origin and variety appear less evident within the products evaluated.

## 1. Introduction

Cocoa is a major food-industrial crop, with global production exceeding 5.02 million tonnes in 2022/23 and an estimated 4.37 million tonnes in 2023/24 [1]. Its cultivation is economically significant for smallholder farmers in tropical regions, providing livelihoods and contributing to regional development [2]. Chocolate, derived from cocoa processing, is valued not only for its sensory properties but also for the nutritional and health-promoting effects associated with its bioactive compounds [3,4]. The global cocoa supply chain, spanning tropical production regions and industrialized processing and consumption centers, presents challenges for product authentication and traceability. The growing demand for sustainable, certified, and single-origin products has driven research into chemical markers capable of discriminating samples according to origin, processing, and quality attributes. Among these, volatiles, lipids, polyphenols, amino acids, and biogenic amines (BAs) have emerged as promising candidates [5,6,7,8,9].

Cocoa composition and quality are influenced by genetic variety, environmental conditions, and post-harvest practices [6,10,11,12,13,14,15]. Fermentation, roasting, and other industrial processing steps generate flavor precursors, pigments, and bioactive compounds, which ultimately define the sensory and chemical profile of chocolate [16,17]. Variability in production systems, from industrial to artisanal, as well as formulation differences, contributes to compositional heterogeneity, highlighting the need for reliable traceability and quality assessment tools. In other food matrices, including wine, cheese, and honey, multivariate analysis of amino acid and BA profiles has been successfully applied to discriminate products according to origin, raw material, or processing conditions [18,19,20]. In cocoa, research has predominantly focused on raw beans, evaluating fermentation dynamics, roasting effects [12,21,22], geographical origin [23], and varietal differences [24]. Applications to finished cocoa-based products are less frequent. Studies by Restuccia and coworkers examined cocoa-based products, including a limited number of chocolate samples, to discriminate among organic, fair-trade, and conventional products [25,26]. Nevertheless, unlike cocoa, chocolate contains various additional ingredients that can also influence the levels of amine compounds, making the interpretation of the results more complex. Other works addressing chocolate have focused on monitoring health-related aspects [27], method development [28,29], or testing specific processing conditions [15,30].

Amino acids and biogenic amines are key indicators of some cocoa features. Proteolysis during fermentation generates free amino acids, which in turn serve as precursors of biogenic amines through the activity of various microorganisms. Some of these compounds also participate in Maillard and Strecker reactions during roasting, contributing to the characteristic aroma and flavor of chocolate [30,31,32]. Levels of both amino acids and BAs are affected by successive processing stages, making them promising markers of distinctive compositional characteristics [33,34,35]. Because these compounds are highly polar, sample preparation represents a critical step in their determination. Different extraction approaches have been reported for cocoa and chocolate matrices, including acidified aqueous media based on perchloric acid, trichloroacetic acid, or formic acid, among others. These acidic media improve analyte extraction while promoting protein precipitation and removal of other insoluble components prior to chromatographic analysis [15,36].

Despite extensive research on raw cocoa, the combined use of LC–MS/MS-based amino acid and biogenic amine profiling with chemometric approaches for the characterization and classification of finished chocolate remains limited [37]. Moreover, studies involving large numbers of commercial samples generally require long LC–MS/MS analytical sequences, during which short- and long-term fluctuations in instrument response may occur, leading to signal drift and increased analytical variability [38,39]. Such effects are particularly critical in multivariate analyses because uncontrolled instrumental drift can mask genuine compositional differences or introduce artificial trends, thereby compromising the interpretation of exploratory and supervised classification models. Therefore, appropriate signal correction and normalization strategies are essential to ensure that chemometric analyses reflect true compositional differences rather than analytical variability. In this context, the influence of these correction procedures on the robustness and interpretability of amino acid and biogenic amine-based chemometric models has received limited attention, particularly in studies addressing finished cocoa products.

The present study addressed two main objectives. First, to establish a robust LC–MS/MS workflow for amino acid and amine profiling by evaluating signal correction and normalization strategies to ensure reliable chemometric analysis, a particularly critical aspect when large sample sets are analyzed in batches over several days. Second, to assess the potential of these compositional profiles to characterize and classify commercial dark chocolates according to botanical variety, geographical origin, cocoa content, and manufacturer.

## 2. Results and Discussion

The reliability of chemometric or machine learning methods depends not only on the representativeness of the dataset but also on the quality of the analytical measurements. Exploratory examination of the raw LC–MS/MS data revealed trends deviating from the compositional patterns expected for the sample set. Such deviations indicate the presence of instrumental drift and temporal variability. These sources of variation can compromise quantification accuracy and potentially lead to misleading patterns in multivariate analyses. This challenge is particularly relevant in LC–MS/MS workflows, where gradual changes in chromatographic performance and ion source stability can introduce systematic drift over long analytical sequences.

To ensure data reliability, different signal correction strategies were evaluated and applied to minimize drift-related artefacts and improve the comparability of responses across the analytical sequence. Once the corrected dataset was established, amino acid and amine profiling enabled the identification of compositional trends. The corrected data matrix was subsequently used for chemometric modelling, allowing the exploration of how cocoa content and other features shape the chemical diversity of dark chocolates.

### 2.1. Data Quality Assessment and Signal Correction

LC-MS/MS is an important tool for quantitative analysis due to its sensitivity, capability, and selectivity, among others. However, signal drift in LC-MS/MS data represents a significant challenge, often arising from gradual changes in chromatographic performance and signal intensity due to contamination build-up on the ion source components of the mass spectrometer [40,41].

**Figure 1:**
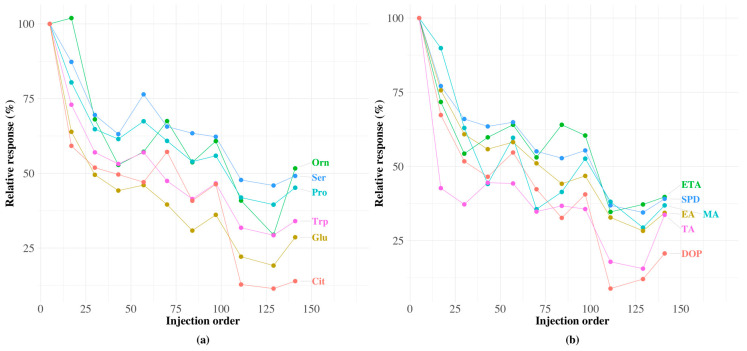
Relative response of selected (**a**) amino acids and (**b**) amines plotted as a function of injection order for QCT samples. Abbreviations: Orn, ornithine; Ser, serine; Pro, proline; Trp, tryptophan; Glu, glutamic acid; Cit, citrulline; ETA, ethanolamine; SPD, spermidine; MA, methylamine; TA, tyramine; EA, ethylamine; DOP, dopamine.

In this study, the analysis of 91 dark chocolate samples and controls provided a representative dataset for examining these stability-related challenges. Since the measurement procedure for samples, standards, QCs, and blanks extends over 120 h, it becomes impossible to maintain signal stability throughout the entire sequence. As indicated in Section 3.4, the system operated continuously throughout the series, without interruptions for cleaning procedures or mass calibration. Consistent with this, extended runs revealed progressive analytical response drift, leading to bias and increased variability, which affected data reliability [40]. To assess signal drift, the relative peak areas of the pooled quality control samples (QCTs) were plotted against the injection order. Figure 1 shows the analytical response variations over time for selected (a) amino acids and (b) amines.

Figure 1 illustrates a clear decreasing trend, indicating a progressive loss of signal over time, with analytes exhibiting signal reductions ranging from 54% to 91%. Among the amino acids, Cit exhibited the greatest decline, with responses falling below 20% of the initial value after ca. 110 injections. For the amines, TA and DOP were the most affected, with signals decreasing to as low as 10%.

Given this pronounced attenuation, the potential relationship between drift magnitude and analyte properties was examined. Despite the analytes spanning a broad spectrum of retention times and molecular masses, no consistent relationship was identified. This finding suggests that signal drift is primarily attributable to systematic instrumental factors, such as ion source contamination and progressive sensitivity decline, rather than analyte-specific influences. These mechanisms impact both early- and late-eluting compounds comparably, thereby complicating efforts to model drift behavior accurately.

As a direct consequence, the dataset demonstrated considerable variability, with uncertainties between 25% and 45%, presenting challenges for accurate quantification and the implementation of multivariate techniques. Trend analysis corroborated the existence of statistically significant drift (*p* < 0.05), reflecting systematic signal changes throughout the analytical run [42].

To correct for these effects, an initial series of regression-based models was evaluated. The resulting coefficients of determination varied from 0.50 to 0.86 for linear, 0.76 to 0.96 for logarithmic, and 0.73 to 0.95 for power regression models, values that indicate relatively strong fits in several cases. Despite these relatively strong fits, the corrections only partially reduced variability up to 50% at best, highlighting that such models do not fully capture all sources of instrumental variation.

As a second approach, traditional correction methods based on nearest quality control (NQC) samples and internal standards (IS) were applied [40]. These strategies proved effective for only about 45% of the compounds, for which uncertainty was reduced by up to 80%. Their limited performance is partly attributable to the wide dynamic range of analyte concentrations, which restricts the uniformity of correction across compounds.

**Figure 2:**
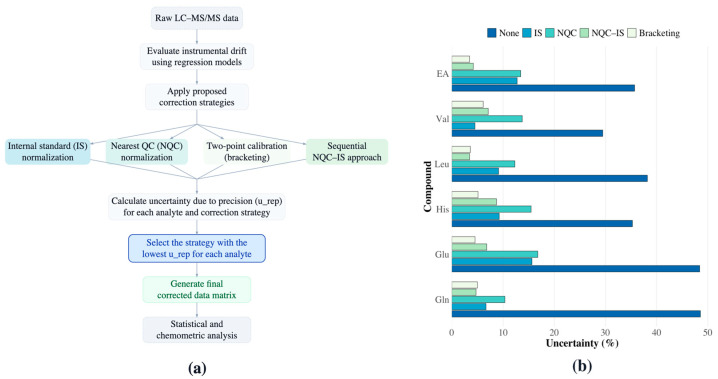
Signal drift correction: (**a**) Workflow for selecting the best correction strategy for each analyte. (**b**) Precision uncertainties obtained for the different signal drift correction approaches. Abbreviations: EA, ethylamine; Val, valine; Leu, leucine; His, histidine; Glu, glutamic acid; Gln, glutamine; IS, internal standard; NQC, nearest quality control; NQC-IS, nearest quality control + internal standard.

For the remaining analytes, more flexible approaches capable of compensating for near real-time instrumental fluctuations and integrating multiple internal standards were subsequently implemented, including bracketing and a combination of NQC and IS normalization. The overall workflow used to evaluate the candidate correction strategies and to select the optimal correction method for each analyte is summarized in Figure 2a. Figure 2b presents the uncertainty values obtained for selected analytes using these correction procedures.

**Figure 3:**
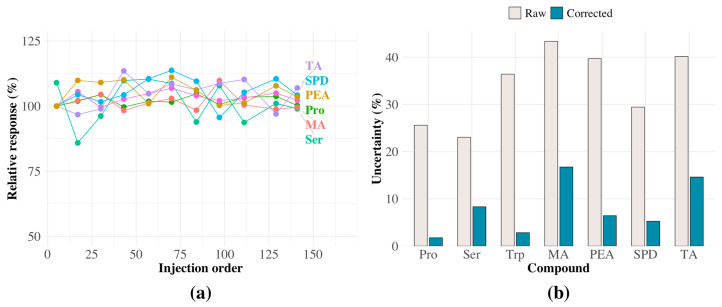
Effect of drift correction: (**a**) Relative response of selected analytes as a function of injection order for QCT samples after correction. (**b**) Uncertainties obtained for raw and corrected data. Abbreviations: Pro, proline; Ser, serine; Trp, tryptophan; MA, methylamine; PEA, phenylethylamine; SPD, spermidine; TA, tyramine.

Figure 2 demonstrates that no single correction method was universally optimal. Bracketing and the combined NQC-IS approach performed best because they accounted for both local instrumental fluctuations and broader temporal drift. This success is attributed to their capacity to account for both local instrument variation, proximate to the sample measurement, and variation occurring during the measurement process itself, in agreement with strategies reported in previous studies. Finally, to maximize precision across all analytes, a hybrid strategy was adopted, selecting for each compound the correction method that yielded the lowest uncertainty. The resulting profiles and uncertainties are presented in Figure 3.

Figure 3 shows that the implementation of these corrections led to substantially improved signal stability, with u_rep_ values decreasing by up to 18-fold and ranging between 1.76% (Pro) and 16.75% (MA) across all compounds. These results are highly relevant, as they ensure that both the concentration profiles and subsequent multivariate analyses are reliable. Furthermore, drift correction impacts both data variability and the capacity of multivariate models to identify underlying chemical patterns and sample-specific trends; this topic will be addressed in detail in the following sections.

### 2.2. Amino Acid and Amine Profiles

**Figure 4:**
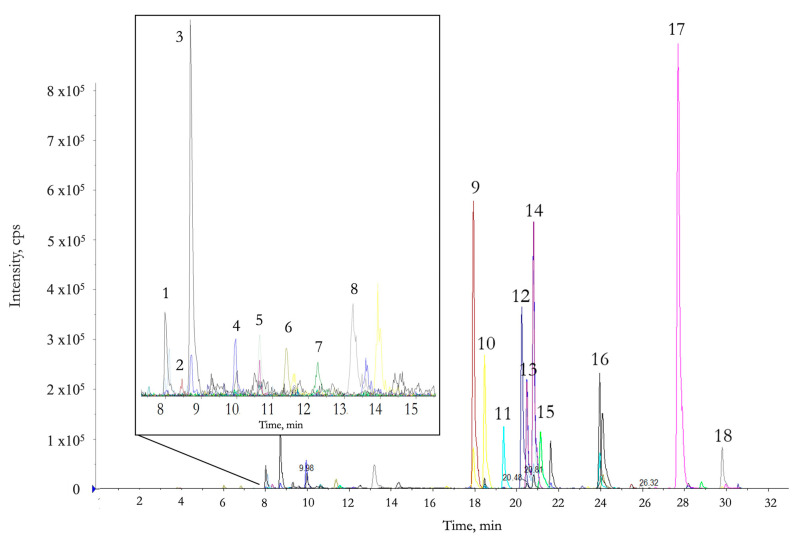
Representative LC–MS/MS total ion chromatogram (TIC) of a chocolate sample (X03), showing detected amino acids and amines. Peak numbers correspond to the following compounds: (1) asparagine, (2) citrulline, (3) serine, (4) methionine, (5) aspartic and glutamic acids, (6) ethanolamine and glycine, (7) alanine, (8) proline, (9) valine, (10) tryptophan, (11) phenylalanine, (12) isoleucine and leucine, (13) ethylamine and hydroxyproline, (14) dimethylamine, (15) butylamine, phenylethylamine and lysine, (16) tyrosine, (17) dopamine and histidine, and (18) tyramine. A zoom for the first region highlights the early elution peaks for better visualization.

From the 38 initially monitored compounds, a total of 27, comprising 19 amino acids and 8 amines, were identified across 91 commercial dark chocolate samples. Figure 4 presents a representative chromatogram of a chocolate sample with the detected amino acids and amines.

Concentrations varied substantially among samples, reflecting differences in cocoa content and cocoa variety. Table 1 and Table 2 summarize the mean concentrations and observed ranges of amino acids and amines, respectively, according to bean variety.

Table 1 shows that the predominant amino acids across all varieties were Tyr, Hyp, Pro, Leu, Phe and Val, with concentrations generally exceeding 700 mg kg^−1^ and accounting for nearly 75% of the total. These results are consistent with previous studies, where hydrophobic amino acids are typically dominant [27], with concentrations for a 70% dark chocolate of the same order of magnitude as those reported here. Leu, Val, and Phe are particularly relevant to cocoa chemistry because their concentrations increase during fermentation, and they act as key precursors in the Maillard reaction during roasting [12,17,44].

Beyond overall abundance patterns, varietal differences were also examined. In contrast, Cit and Orn were detected at low levels in ca. 80% of samples, typically below 10 mg kg^−1^, contributing less than 1%, and Met was not detected in any sample. All remaining amino acids accounted for less than 3%. Although amino acids and amines are key precursors of flavor-active compounds, their concentrations in finished chocolate cannot be directly interpreted as sensory attributes.

Although the magnitude of variation was limited, some varietal trends were detectable. Criollo and Trinitario samples tended to display higher overall amino acid contents, particularly for aromatic and hydrophobic amino acids (Tyr, Phe, and Leu). Conversely, Forastero and Nacional samples tended to show lower total amino acid levels but relatively higher Ala and Cys contents. Despite these apparent trends, statistical testing using Tukey’s HSD indicated that most differences were not significant (*p* > 0.05) (Figure S1, Supplementary Material).

Table 2 summarizes the concentrations for amines, indicating that the polyamine SPD was the most prevalent, representing 26% of the total. This was followed by HTA and EA (~16% each), MA and PEA (~10% each), DOP and DMA (~8% each), and TA (2.8%). Compounds such as SPM, PUT, and CAD, although reported in other studies [21,26,28,45], were barely detected in the samples.

Among cocoa varieties, Forastero samples showed the highest concentrations of several aliphatic amines. DMA reached 781 mg kg^−1^ and EA 391 mg kg^−1^, compared with 415 mg kg^−1^ (DMA) and 204 mg kg^−1^ (EA) in Trinitario. Such enrichment of low-molecular-weight aliphatic amines in Forastero is consistent with more extensive microbial decarboxylation associated with longer or more intense fermentation [24]. In contrast, Criollo samples exhibited relatively higher levels of aromatic and polyamine species: PEA averaged 23.1 mg kg^−1^, MA 25.7 mg kg^−1^, and SPD 49.8 mg kg^−1^ (Table 2). Blend and Trinitario chocolates also contained appreciable amounts of aromatic amines and HTA (e.g., HTA: Blend 30.9 mg kg^−1^; Trinitario 27.6 mg kg^−1^), whereas Nacional samples displayed intermediate concentrations for most compounds (e.g., DMA 530 mg kg^−1^; EA 262 mg kg^−1^; SPD 35.3 mg kg^−1^).

Overall, the results are consistent with previous reports on cocoa-derived products, including the comprehensive survey by Esposito and coworkers [46], who analyzed 59 cocoa powder samples from the European market and confirmed the generally low levels of amines. However, the inherent heterogeneity of cocoa matrices, combined with the overlapping effects of fermentation, roasting, and formulation, makes it difficult to draw definitive conclusions from individual compounds alone. These complexities underscore the need for multivariate approaches to capture the underlying compositional patterns and classify samples according to cocoa content, variety, and processing-related factors.

### 2.3. Exploratory Analysis of Chocolate Samples

Consistent with the drift patterns described above, instrumental drift can introduce systematic variability in the dataset. This effect was clearly reflected when PCA was applied to the raw data, including QC samples. The resulting score plot exhibited substantial dispersion among QCs and a pronounced trend along PC1 as a function of injection order, highlighting how drift can obscure true chemical differences and affect the interpretation of sample distributions in chemometric analyses. In the uncorrected dataset, PC1 captured approximately 65% of the total variance, largely reflecting the injection-order trend associated with instrumental drift, while PC2 explained around 12%. This distribution of variance confirms that temporal drift dominated the multivariate structure of the raw data, as shown in the comparative PCA results provided in the Supplementary Material (Figure S2a).

PCA was then performed on the data matrix after applying the selected correction strategy for each compound. In this second model, QC samples appeared tightly clustered, demonstrating that the correction strategies effectively mitigated instrumental drift, substantially reduced analytical variability among QC injections, and improved overall data quality. Under these circumstances, the corrected matrix provided a reliable basis for subsequent multivariate analysis. Accordingly, the PLS-DA models were constructed using the corrected data matrix, ensuring that model interpretation was driven by compositional differences among samples rather than instrumental effects. Comparative score plots before and after correction are provided in the Supplementary Material (Figure S2).

**Figure 5:**
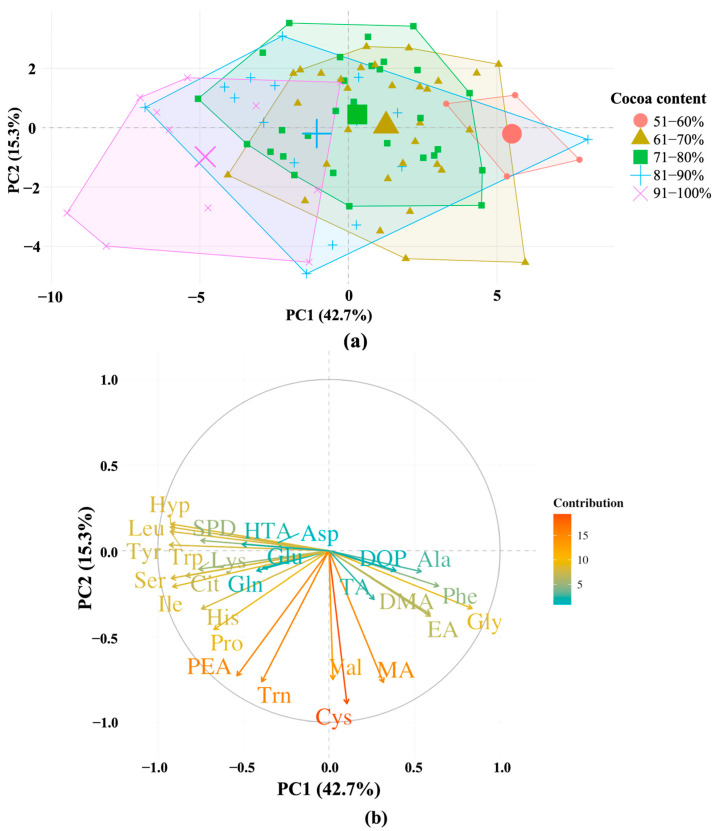
Principal Component Analysis (PCA) of 91 commercial chocolate samples based on the relative content of amino acids and amines. (**a**) Scores plot of the first two principal components (PC1 and PC2). (**b**) Loading plot showing the contribution of each variable to the sample distribution. Abbreviations: Gly, glycine; Phe, phenylalanine; EA, ethylamine; DMA, dimethylamine; Ala, alanine; DOP, dopamine; TA, tyramine; MA, methylamine; Cys, cysteine; Val, valine; Trn, threonine; PEA, phenylethylamine; Pro, proline; His, histidine; Ile, isoleucine; Ser, serine; Tyr, tyrosine; Trp, tryptophan; Hyp, hydroxyproline; Leu, leucine; Cit, citrulline; Lys, lysine; SPD, spermidine; Gln, glutamine; Glu, glutamic acid.

PCA excluding QCs was used to examine patterns in the sample distribution. The first two PCs explained nearly 60% of the total variance (Figure 5). This value suggests that the dataset is complex and that, as previously noted, multiple factors may influence the concentrations of amines and amino acids in the samples. Furthermore, it should be noted that this plot represents only a partial projection of the information, and some patterns may be captured in combinations of higher-order PCs.

No clear patterns were observed in this unsupervised analysis when samples were evaluated based on declared features, such as origin, cocoa variety, or certification schemes such as Fairtrade, organic, and single origin, highlighting the complexity of commercial chocolate matrices [16,47]. Unlike raw cocoa beans, commercial chocolates are formulated products whose composition is influenced not only by cocoa origin and variety but also by cocoa percentage, formulation, and manufacturing practices. Although the declared cocoa percentage reflects the total proportion of cocoa-derived ingredients, chocolates with similar cocoa percentages may differ in the relative proportions of cocoa liquor and cocoa butter, as well as in minor formulation components. Beyond declared attributes, formulation and processing also contribute to the overall variability of commercial chocolates and may partially mask the more subtle compositional differences associated with cocoa origin or variety, thereby limiting their discrimination by unsupervised PCA.

Among the evaluated attributes, cocoa percentage showed the strongest and most consistent relationship with sample distribution. The score plot presented in Figure 5a revealed a broad dispersion of samples, without compact clusters corresponding to well-defined classes. These compositional tendencies are consistent with the combined influence of cocoa raw materials, fermentation, roasting, and formulation practices, although the specific contribution of each factor cannot be independently assessed with the available information.

Instead, a continuous compositional trend associated with cocoa content was observed along PC1. Chocolates with lower cocoa percentages (51–70%) were positioned on the positive side, whereas samples with higher cocoa content (81–100%) appeared on the negative side. The centroids of each class, represented by the larger symbols located at the center of each group, further support this gradual trend. This pattern reflects the direct relationship between cocoa mass and nitrogenous compounds: higher cocoa content corresponds to increased levels of proteins and free amino acids, which serve as precursors of further amines. PC2 may capture other, more complex sources of variance or combinations of factors, and its interpretation is therefore not straightforward.

The corresponding loading plot (Figure 5b) highlights the variables contributing most to the variance captured by PC1. Some analytes played a decisive role in discriminating between samples with low and high cocoa content. Compounds such as Pro, His, Tyr, PEA, and SPD were more abundant in high-cocoa samples, whereas analytes with positive PC1 loadings (e.g., EA, Gly, and Phe) were more prevalent in low-cocoa samples. These compounds can be associated with milder formulations, where lower cocoa content and higher proportions of added ingredients dilute the nitrogenous fraction [48]. Overall, the PCA indicates that cocoa percentage is the most visible attribute associated with amino acid and amine composition. However, the broad overlap among samples indicates that additional sources of variability also contribute to the chemical profiles of commercial chocolates, limiting discrimination according to cocoa origin or variety.

### 2.4. Sample Classification by Partial Least Squares-Discriminant Analysis (PLS-DA)

To evaluate the classification potential of the measured amino acids and amines, PLS-DA models were constructed. Due to the limited number of samples available for the different typologies, independent model validation was not feasible. Instead, model performance was assessed using a repeated random-block cross-validation strategy (see Section 3.6). This study was complemented by a permutation test to provide a robust evaluation of the classification performance and demonstrate that the observed classification accuracy is significantly higher than would be expected from random assignments (see below). Hence, although the application conditions for PLS-DA were not optimal due to this limited representativity, PLS-DA offers a good compromise between predictive performance and model interpretability, reducing the risk of overfitting compared with more complex machine-learning methods and facilitating the identification of the chemical features driving class separation.

Initial classification attempts based on cocoa variety, origin, and certification yielded weak discrimination, similar to PCA results. In contrast, manufacturer-related attributes produced more distinct sample separation. The limited discrimination observed for the remaining classifications likely reflects the complexity of commercial chocolate matrices, where multiple factors simultaneously influence amino acid and amine profiles. In addition, the variability introduced during industrial processing and formulation may partially mask compositional signatures associated with cocoa origin or variety. Within the commercial chocolates evaluated in this study, manufacturer-related factors appeared to contribute more strongly to amino acid and amine variability than the declared geographical or botanical origin of the cocoa beans.

To reduce class imbalance, which can bias model performance, the analysis was restricted to a subset of major brands (with at least five samples, i.e., Blanxart, Kallari-Yumbos, Sprüngli, Tibito, and Xocolata Jolonch). Model optimization was performed through repeated M-fold cross-validation, which determined the optimal number of components and the most discriminant variables by minimizing the balanced error rate (BER). Different prediction distances were tested (centroid, maximum, and Mahalanobis), with the Mahalanobis distance consistently yielding the lowest error rates. The final model, based on three latent variables that collectively explained 75% of the total variance, achieved a cross-validated balanced classification accuracy of 75.2% (BER = 0.248). The robustness of the optimized model was further assessed by a permutation test (1000 permutations). None of the permuted models achieved a cross-validated balanced classification accuracy equal to or greater than that of the original model, indicating that the observed discrimination was highly unlikely to arise by chance (*p* < 0.001). The distribution of balanced classification accuracies obtained from the permutation test is shown in Figure S3.

The predictive performance obtained should be interpreted considering the complexity of the classification task. In contrast to studies focused on the discrimination of cocoa varieties or geographical origins using relatively homogeneous materials, the present work addresses commercial dark chocolates, whose amino acid and amine profiles are simultaneously influenced by multiple sources of variability, including cocoa variety, geographical origin, cocoa content, fermentation, roasting, formulation, and the frequent use of blended cocoa beans. Consequently, partial overlap between manufacturers is expected, making brand classification substantially more challenging. Within this context, the balanced classification accuracy achieved by the optimized PLS-DA model indicates that amino acid and amine profiles retain sufficient manufacturer-specific information to support meaningful discrimination despite the complexity of the samples.

**Figure 6:**
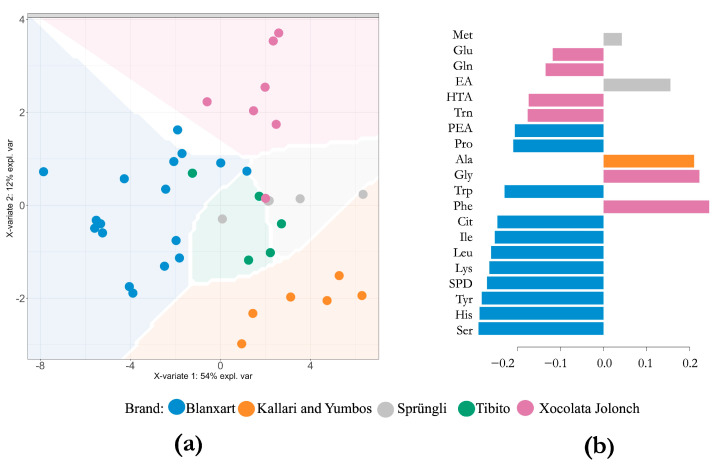
PLS-DA of chocolate samples. (**a**) Score plot with Mahalanobis prediction background showing sample clustering by manufacturer. (**b**) Loading plot highlighting the variables driving the separation. Colours indicate the brand with the highest median value for each compound. Abbreviations: Met, methionine; Glu, glutamic acid; Gln, glutamine; EA, ethylamine; HTA, hydroxytryptamine; Trn, threonine; PEA, phenylethylamine; Pro, proline; Ala, alanine; Gly, glycine; Trp, tryptophan; Phe, phenylalanine; Cit, citrulline; Ile, isoleucine; Leu, leucine; Lys, lysine; SPD, spermidine; Tyr, tyrosine; His, histidine; Ser, serine.

Figure 6a shows the score plot of the optimized model with a Mahalanobis prediction background. The first two latent components explained 54% and 12% of the total variance, respectively. The coloured background represents the prediction areas for each class based on Mahalanobis distance, whereas the white regions correspond to unassigned areas where the model did not reach a confident classification. Overall, three manufacturers formed distinct clusters, whereas two showed partial overlap. Blanxart (blue) formed a well-defined cluster on the left side of the plot, while Kallari and Yumbos (orange) grouped tightly in the lower right quadrant. Xocolata Jolonch (pink) occupied the upper region of the space with a distinct separation from the other classes. In contrast, Sprüngli (gray) and Tibito (green) were positioned closer to the plot center, showing partial overlap with other classes, likely due to sharing characteristics with other groups and exhibiting less pronounced manufacturer-specific compositional patterns.

The variable contribution plot on LV1 (Figure 6b) highlights the compounds contributing most strongly to the separation among manufacturer-related groups. Among the most influential variables were the amino acids Ser, His, Tyr, Lys, Leu, Ile, and Cit, together with the amines SPD and PEA. The loading structure revealed differential associations between sample groups and specific analytes. For example, samples from Kallari–Yumbos were associated with higher contributions of Ala along LV1, whereas Xocolata Jolonch samples were associated with Phe, Gly, and HTA, and showed opposite contributions from Trn, Gln, and Glu. Additional contributions captured by LV2 and LV3 (Figure S4) involved compounds such as Val, Cys, Gln, Phe, Met, and HTA for Xocolata Jolonch and Sprüngli. Blanxart samples showed moderate associations with Pro, Leu, Tyr, Ser, SPD, and PEA, whereas EA contributed more strongly to the discrimination of the Sprüngli group along LV3. While these associations highlight compositional tendencies, they should be interpreted as patterns rather than definitive markers. Because detailed information regarding fermentation, roasting, and formulation was unavailable, the contribution of individual processing factors cannot be independently determined. Nevertheless, the reproducibility of these patterns across samples suggests that manufacturer-related practices contribute measurably to the amino acid and amine fingerprints of commercial chocolates.

**Figure 7:**
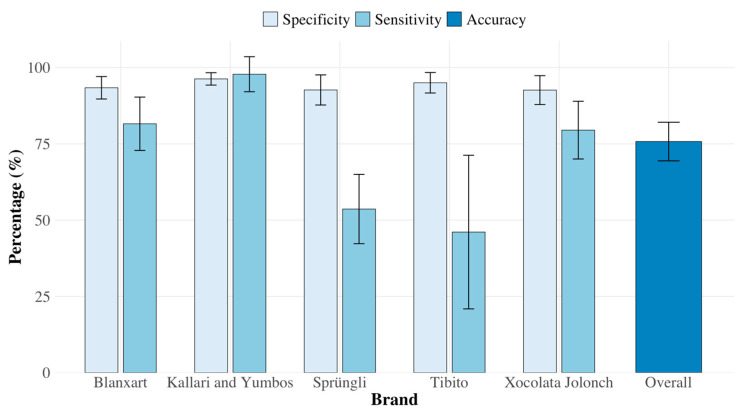
Performance metrics of the classification of the PLS-DA classification model for chocolate samples.

Figure 7 summarizes the class-specific sensitivity and specificity obtained during repeated cross-validation, with error bars representing variability across the 20 cross-validation repetitions. Sensitivity ranged from 49% for Tibito to 97.5% for Kallari–Yumbos, whereas specificity remained consistently high across all classes (92.5–96.3%). The lower sensitivity observed for Sprüngli and Tibito is consistent with their broader dispersion and partial overlap in the score plot, indicating greater within-group heterogeneity and consequently more challenging classification.

Differences in amino acid and amine profiles are likely influenced by multiple factors, including cocoa raw materials, fermentation, roasting, and formulation practices. Among these processes, roasting plays a particularly important role because amino acids act as precursors in Maillard and Strecker reactions, contributing to the formation of numerous flavor-active compounds [44,49]. The abundance of hydrophobic amino acids such as Leu, Phe, Tyr, and Val may therefore reflect not only the composition of the cocoa material but also the cumulative effects of post-harvest processing and chocolate manufacture [17]. Subsequent operations, including conching and formulation adjustments, may further modify the relative abundance of these compounds and contribute to the compositional variability observed among commercial products [15,50].

This diversity of sources of variability also helps contextualize the classification performance of the PLS-DA model. When interpreted within this multifactorial context, the balanced classification accuracy achieved by PLS-DA aligns with values reported in the literature for supervised chemometric models applied to other complex processed food matrices, where overlapping characteristics commonly limit the predictive accuracy [20,46,51,52]. By contrast, substantially higher accuracies are generally observed in studies involving well-defined botanical varieties or controlled experimental materials, where the number of confounding factors is considerably lower [6,12,22,23]. Taken together, these factors help explain the chemical differences captured by the PLS-DA model.

## 3. Materials and Methods

### 3.1. Chemicals

A total of 38 analytical standards (purity > 95%) corresponding to amino acids and amines were used in this study. Detailed information, including compound supplier and intended use (target analytes, surrogate standards, and instrumental internal standards), is provided in Table S1 (Supplementary Material).

Hexylamine (HEX) hydrochloride and butylamine (BUT) hydrochloride (TCI, Tokyo, Japan) were used as surrogate standards, added prior to derivatization and served to monitor derivatization consistency across the sample measurement. L-asparagine (Asn, Carlo Erba, Milan, Italy), isopropyl amine (IPA, TCI, Tokyo, Japan) hydrochloride, and octopamine (OCT, TCI, Tokyo, Japan) were used as Instrumental Internal Standards in the way that DNS-derivatives were added after derivatization to compensate for signal drift and ionization efficiency variations during the extended LC-MS/MS sequences.

Standard stock solutions were prepared from the analytical standards listed in Table S1 at concentrations of 1000 mg L^−1^ in 0.2 M hydrochloric acid and stored in amber glass vials at 4 °C until use.

LC-MS grade acetonitrile and acetone (ACS-grade) from Sharlab (Barcelona, Spain) and ultrapure water produced by a Milli-Q system (Merck, Darmstadt, Germany) were used as solvents, while LC-MS grade formic acid was used as mobile phase modifier (Merck, Darmstadt, Germany). Dansyl chloride (DNSCl) 98% from Acros Organics (Geel, Belgium) was used as the derivatizing reagent. Sodium carbonate, sodium hydroxide, ammonium hydroxide, and hydrochloric acid (37%) were obtained from Sigma Aldrich (Steinheim, Germany).

### 3.2. Samples

A total of 91 dark chocolate bar samples representing 31 commercial brands were included in the study, with declared cocoa contents ranging from 56% to 100%. The chocolates were manufactured across 10 countries and 23 cities across Europe, the Americas, and Australia, using cacao beans sourced from countries across Africa, Asia, Oceania, and Central and South America.

Sample distribution according to bean origin (continent), declared genetic variety, certification status, and cocoa content is summarized in Table 3. Individual sample information is provided in the Supplementary Material (Table S2).

Chocolate samples were purchased over the course of the study rather than during a single sampling campaign. After purchase, samples were stored under controlled temperature and light-protected conditions and analyzed prior to their manufacturer-declared best-before dates (typically 2025–2026). All sample characteristics, including variety, geographical origin, and certification status, were obtained directly from product label information, supplemented with data from company websites.

### 3.3. Sample Pretreatment

The extraction of amino acids and amines was performed following the procedure described by Delgado-Ospina et al., [15] with modifications for the chocolate matrix. Briefly, chocolate bars were first frozen and ground to a homogeneous powder. A 1 g ± 0.01 g portion of each sample was mixed with 10 mL of 0.2 M hydrochloric acid and vortexed for 30 s. Extractions were carried out in a water bath at 60 °C ± 2 °C for 30 min under constant magnetic stirring. Surrogate standards (HEX and BUT at ca. 10 mg L^−1^) were added here. Extracts were centrifuged at 4500 rpm for 10 min, and the supernatant was separated and reserved for derivatization.

Derivatization was performed on a 250 µL aliquot of the extract, following the protocol developed by Navarro-Abril et al. [53] with adaptations. First, the pH was adjusted to approximately 10.6 with the addition of 250 µL of 215 mM sodium hydroxide/385 mM sodium carbonate solution, and then 250 µL of a 12 mg mL^−1^ DNSCl solution was added for derivatization. The reaction proceeded in the dark at 45 °C ± 2 °C for 30 min. The excess of DNSCl was eliminated by adding 40 µL of ammonium hydroxide (25%, *w*/*v*), vortexing for 1 min, and keeping in the dark for 10 min. Finally, a mixture of Asn, OCT, and IPA DNS derivatives at ca. 10 mg L^−1^ was added as instrumental internal standards. Derivatized extracts were filtered through a 0.45 µm nylon syringe filter and analyzed by LC-MS/MS.

### 3.4. LC-MS/MS Analysis

The analytical determination of DNS-derivatized amino acids and amines was performed using an Agilent 1100 Series LC system (Agilent Technologies, Palo Alto, CA, USA) coupled to an AB Sciex 4000 QTrap hybrid triple quadrupole/linear ion trap mass spectrometer (AB Sciex, Framingham, MA, USA). LC–MS/MS conditions were based on a methodology previously established by the research group [53,54], with minor adaptations for the chocolate matrix.

Chromatographic separation was achieved on a Kinetex^®^ C18 column (100 mm × 4.6 mm, 2.6 µm particle size; Phenomenex, Torrance, CA, USA) operated at room temperature. The mobile phase consisted of 0.1% formic acid in water (A) and acetonitrile (B). The gradient started at 5% B, increased linearly to 24% B over 7 min, and was held at 24% B for 5 min, reached 40% B at 16 min, 70% B at 28 min, and 100% B at 29 min, maintained for 1 min, then returned to 5% B with a 2 min re-equilibration period. The flow rate was maintained at 850 µL min^−1^, and the injection volume was 5 µL.

MS/MS detection was performed in multiple reaction monitoring (MRM) mode using an electrospray ionization (ESI) source operating in positive polarity at 4500 V. Nitrogen was employed as ion source gas 1 and 2 (both at 50 a.u.) and as curtain gas (20 psi). Compound-specific precursor ions (Q1), product ions (Q3), declustering potentials (DP), collision energies (CE), and collision cell exit potentials (CXP [53]) are summarized in Table S3. Each precursor (Q1) and product ion (Q3) pair was acquired at unit resolution using a dwell time of 20 ms per transition. Instrument control, data acquisition, and analysis were performed using Analyst^®^ software, version 1.6.2 (AB Sciex, Framingham, MA, USA).

Chocolate samples were extracted and derivatized in triplicate and analyzed by LC–MS/MS in random order to minimize potential systematic errors. No instrument maintenance, source cleaning, or mass spectrometer recalibration was performed during the analytical sequence to preserve consistent instrumental conditions throughout the entire run. Throughout the injection sequence, blank and quality control (QC) samples were periodically analyzed to evaluate the stability of the system. The blank consisted of a mixture of acetonitrile and water, which was injected regularly—every 15 samples—to verify the absence of carryover. Two types of QC samples were employed: a composite sample prepared by pooling 100 µL from each extract (here referred to as QCT) and a commercial chocolate sample measured repeatedly across the sequence (QMR); in the two cases, every 15 samples, we performed injections.

QCT is an average pool of all samples. This type of QC sample is commonly used in chemometric studies to evaluate data stability and to verify its central position within PCA models without trends associated with the injection order. The QMR is a completely independent chocolate sample to serve as an external control to evaluate the effectiveness of the applied corrections; the precision of the QMR values after the treatment is an objective indication of the quality of the correction strategies.

Analytical performance characteristics relevant to the objectives of the present study were evaluated after adaptation of the method to the chocolate matrix to confirm its suitability for the intended application. Since the study focused on compositional fingerprinting rather than absolute quantification, linearity and repeatability were evaluated for representative analytes covering different chemical classes. Two surrogate standards (hexylamine and butylamine) were added prior to derivatization to monitor the consistency of the derivatization procedure. No recovery correction was applied, as relative LC–MS/MS peak areas were used for subsequent data analysis. The corresponding analytical performance characteristics are summarized in Table S4.

### 3.5. Data Processing and Correction

LC–MS/MS peak areas of amino acids and amines were corrected to minimize instrumental drift. To improve the data quality prior to multivariate exploratory and classification analyses, we assessed several options that potentially could meet our needs, which arise directly from our own research. The evaluation of preprocessing performance relies on identifying the protocol that provides the best reproducibility in the estimated concentrations of the QC sample, as these injections were distributed regularly throughout the analytical sequence. A complementary criterion considers the whole analytical workflow up to the construction of the chemometric models, in which the QC samples should appear tightly clustered and free from trends associated with time or injection order.

In this context, four correction strategies were evaluated: an internal standard normalization (IS), in which the raw signal of each analyte was normalized to the most strongly correlated candidate IS (Asn, OCT, and IPA); a nearest quality control normalization (NQC) method that normalized sample responses using the QC injection acquired closest in time, compensating for short-term instrumental fluctuations; a two-point calibration (bracketing) procedure based on linear interpolation between the QC injections immediately preceding and following each sample, using a correction factor defined as the ratio between the median QCT response and the interpolated QC value; and a combined NQC–IS strategy that sequentially applied IS normalization followed by NQC correction to integrate compound-specific and temporal adjustments. The performance of each correction strategy was evaluated by the uncertainty due to precision (u_rep_), estimated using the QMR sample.

### 3.6. Statistical Analysis

A data matrix of 91 samples by 27 target compound LC-MS/MS peak areas was built using the corresponding average values for each sample. The corrected matrix was then autoscaled to standardize the variables. Principal Component Analysis (PCA) was first applied as an unsupervised exploratory method.

For the PLS-DA, the autoscaled matrix of compositional data was related to several attributes, such as variety, cocoa content, and brand, using a multiclass modeling approach. PLS-DA was selected because it is particularly suitable for highly correlated compositional datasets, allowing simultaneous dimensionality reduction, supervised classification, and interpretation of the variables contributing to class discrimination. Sample assignment to a given class was performed using the maximum probability rule, thereby avoiding non-assignments or multiple assignments. Model optimization was conducted using repeated M-fold cross-validation (20 repetitions, 3 folds), and the optimal number of latent variables (LVs) was selected by minimizing the balanced error rate (BER). Different prediction distances, including centroid, maximum, and Mahalanobis distance, were evaluated.

Model performance was assessed in terms of sensitivity, specificity, and accuracy. Sensitivity was calculated as the ratio between samples correctly assigned to a class and the total number of samples belonging to that class; specificity was computed as the ratio between samples correctly recognized as not belonging to the class and the total number of out-of-class samples; and prediction error was defined as the ratio of misclassified samples (under any circumstance) to the total number of samples. To evaluate whether the observed classification performance exceeded that expected by chance, a permutation test (1000 permutations) was performed using the optimized PLS-DA workflow.

All analyses were conducted in RStudio (Version 2024.06.14) using the R environment [55]. The Factoextra package (version 2.0.0) was used for PCA visualization [56], while the mixOmics package (version 6.30.0) was employed for PLS-DA modeling, cross-validation, and evaluation of classification metrics [57].

Further methodological details can be found in Ballabio et al. and Jiménez-Carvelo et al. [51,52].

## 4. Conclusions

This study provides a comprehensive characterization of amino acid and amine profiles across a large set of commercial chocolates, revealing substantial chemical variability associated primarily with cocoa content and manufacturer-related processing practices. Chocolates produced by the same manufacturer tended to exhibit similar compositional patterns, suggesting that manufacturer-related factors contribute substantially to the observed variability.

Prior to multivariate analysis, instrumental drift was carefully evaluated and corrected, which markedly improved data quality and ensured reliable interpretation of chemometric outcomes. The results showed that the optimal signal-correction approach may vary among analytes. Nevertheless, internal-standard normalization and quality control–based correction, either applied individually or in combination, generally led to significant improvements in data quality and analytical precision. The unsupervised PCA reveals the natural trends among the chocolates, indicating that cocoa content is an influential factor in the distribution of the samples. Although the separation is not complete and a substantial overlap exists between the categories defined by cocoa content, a reasonable progression in sample distribution can nevertheless be observed as a function of this attribute. The lack of a clearer separation is attributed to the complexity and multifactorial nature of the analyte profiles.

Supervised PLS-DA further revealed brand-specific chemical signatures. While some manufacturers displayed highly consistent profiles, enabling clear sample discrimination, others exhibited partial overlap, reflecting variability and shared characteristics with other groups. Altogether, these findings indicate that amino acid and biogenic amine profiles are driven predominantly by cocoa content and processing-related factors. While cocoa origin and variety may contribute to the observed variability, their influence appears to be partially masked in commercial chocolates by industrial processing and formulation practices. This work represents one of the largest evaluations of amino acids and biogenic amines in commercial dark chocolates to date and demonstrates their usefulness as compositional descriptors of chocolate products. Nevertheless, additional studies are required to reinforce these findings and to allow their broader generalization, particularly in order to evaluate other potentially relevant aspects such as quality assurance and product authentication in the chocolate industry.

## Figures and Tables

**Table 1:** Mean concentrations of amino acids (mg kg^−1^) in commercial dark chocolate samples according to cocoa variety. Data are expressed as mean ± standard deviation (SD). Values in parentheses indicate the observed concentration ranges (minimum–maximum).

Compound *	Trinitario	Criollo	Forastero	Nacional	Blend
Asp	(2.8 ± 1.3) × 10^2^ (98.7–618)	(3.0 ± 2.1) × 10^2^ (124–657)	(2.1 ± 0.9) × 10^2^ (111–272)	(2.1 ± 1.8) × 10^2^ (67–873)	(2.7 ± 1.3) × 10^2^ (107–465)
Glu	21 ± 11 (3.7–38.5)	22 ± 23 (9.2–62.7)	19 ± 7 (12.8–26.8)	14 ± 10 (3.5–40.5)	29 ± 18 (10.1–54.8)
Ala	57 ± 16 (32.2–87.8)	63 ± 24 (47.4–105)	74 ± 30 (53.8–108)	70 ± 17 (38.4–96.6)	78 ± 24 (52.8–123)
Cys	13.3 ± 7.2 (5.8–29.0)	23 ± 24 (3.9–65.1)	12.0 ± 5.1 (8.6–17.9)	14 ± 13 (2.9–59.1)	21 ± 16 (4.3–45.8)
Cit	24 ± 31 (0.4–120)	57 ± 91 (0.4–216)	14 ± 12 (0.4–22.8)	23 ± 49 (0.4–207)	28 ± 22 (0.4–54.4)
Phe	(8.4 ± 3.7) × 10^2^ (318–1700)	(8.8 ± 5.4) × 10^2^ (369–1770)	(7.4 ± 2.7) × 10^2^ (465–1010)	(6.7 ± 4.5) × 10^2^ (282–2251)	(9.2 ± 4.0) × 10^2^ (400–1537)
Gly	(4.0 ± 1.1) × 10^2^ (242–560)	(4.2 ± 1.4) × 10^2^ (236–626)	476 ± 8 (467–482)	(4.1 ± 1.1) × 10^2^ (276–692)	(4.7 ± 1.6) × 10^2^ (250–696)
Gln	(6.3 ± 3.1) × 10^2^ (164–1129)	(6.7 ± 6.2) × 10^2^ (321–1758)	(6.0 ± 1.9) × 10^2^ (405–788)	(4.4 ± 2.8) × 10^2^ (153–1201)	(8.8 ± 4.9) × 10^2^ (353–1587)
Hyp	(2.08 ± 0.87) × 10^3^ (851–4187)	(2.2 ± 1.3) × 10^3^ (1073–4409)	(1.62 ± 0.56) × 10^3^ (978–2033)	(1.6 ± 1.1) × 10^3^ (613–5362)	(2.10 ± 0.81) × 10^3^ (938–3247)
His	77 ± 33 (26.6–141)	83 ± 58 (25.0–179)	62 ± 35 (27.5–96.6)	72 ± 49 (17.3–212)	70 ± 25 (24.8–103)
Ile	(2.1 ± 1.1) × 10^2^ (58.4–512)	(2.4 ± 2.2) × 10^2^ (75.7–620)	(1.4 ± 0.6) × 10^2^ (77.6–194)	(1.6 ± 1.8) × 10^2^ (41.2–831)	(2.3 ± 1.2) × 10^2^ (83.8–442)
Leu	(1.06 ± 0.44) × 10^3^ (432–2115)	(1.1 ± 0.7) × 10^3^ (525–2278)	(8.2 ± 2.9) × 10^2^ (493–1036)	(8.2 ± 5.6) × 10^2^ (313–2767)	(1.07 ± 0.42) × 10^3^ (484–1715)
Lys	6.0 ± 2.8 (0.1–12.0)	9 ± 8 (3.0–22.5)	6.1 ± 1.3 (4.7–7.3)	6.0 ± 3.2 (1.6–14.8)	6.9 ± 3.2 (2.3–13.7)
Orn	1.9 ± 1.4 (0.2–4.7)	4.5 ± 2.9 (0.2–7.9)	4.8 ± 5.2 (0.2–10.4)	4.2 ± 3.9 (0.2–11.6)	1.9 ± 1.4 (0.2–4.1)
Pro	(1.28 ± 0.46) × 10^3^ (645–2560)	(1.35 ± 0.57) × 10^3^ (772–2141)	(1.3 ± 0.6) × 10^3^ (856–2006)	(1.2 ± 0.6) × 10^3^ (337–2923)	(1.45 ± 0.45) × 10^3^ (960–2073)
Ser	(3.6 ± 2.0) × 10^2^ (121–929)	(4.8 ± 4.1) × 10^2^ (134–1165)	(2.3 ± 0.9) × 10^2^ (121–298)	(2.9 ± 3.0) × 10^2^ (84.9–1395)	(3.3 ± 1.1) × 10^2^ (175–478)
Tyr	(2.8 ± 1.1) × 10^3^ (1016–4850)	(2.8 ± 1.4) × 10^3^ (1164–4952)	(2.28 ± 0.83) × 10^3^ (1377–3021)	(2.2 ± 1.2) × 10^3^ (625–5506)	(2.70 ± 0.82) × 10^3^ (1312–3705)
Trn	(1.6 ± 0.9) × 10^2^ (50.4–403)	(1.9 ± 1.7) × 10^2^ (55.7–493)	(1.1 ± 0.5) × 10^2^ (55.4–147)	(1.2 ± 1.3) × 10^2^ (33.9–609)	(1.6 ± 0.7) × 10^2^ (58.6–269)
Trp	(1.14 ± 0.46) × 10^2^ (43.9–215)	(1.1 ± 0.7) × 10^2^ (58.1–231)	92 ± 37 (57.3–131)	90 ± 56 (38.0–286)	(1.3 ± 0.5) × 10^2^ (52.9–218)
Val	(8.8 ± 3.6) × 10^2^ (347–1829)	(9.8 ± 6.8) × 10^2^ (387–2119)	(7.0 ± 2.9) × 10^2^ (360–907)	(6.7 ± 5.3) × 10^2^ (246–2567)	(8.6 ± 3.0) × 10^2^ (437–1297)

(*) Abbreviations: Asp, aspartic acid; Glu, glutamic acid; Ala, alanine; Cys, cysteine; Cit, citrulline; Phe, phenylalanine; Gly, glycine; Gln, glutamine; Hyp, hydroxyproline; His, histidine; Ile, isoleucine; Leu, leucine; Lys, lysine; Orn, ornithine; Pro, proline; Ser, serine; Tyr, tyrosine; Trn, threonine; Trp, tryptophan; Val, valine. Values in scientific notation are expressed as (mean ± SD) × 10^n^ according to ISO GUM [43].

**Table 2:** Mean concentrations of amines (mg kg^−1^) in commercial dark chocolate samples according to cocoa variety. Data are expressed as mean ± standard deviation (SD). Values in parentheses indicate the observed concentration ranges (minimum–maximum).

Compound *	Trinitario	Criollo	Forastero	Nacional	Blend
DMA	(4.2 ± 1.4) × 10^2^ (217–743)	(5.3 ± 2.3) × 10^2^ (276–871)	(7.8 ± 3.6) × 10^2^ (388–1096)	(5.3 ± 2.0) × 10^2^ (283–920)	(5.5 ± 1.9) × 10^2^ (291–828)
DOP	12 ± 8 (1.6–25.8)	15 ± 16 (0.6–42.0)	12.6 ± 2.5 (9.8–14.3)	13 ± 10 (2.0–49.3)	13 ± 8 (3.1–26.5)
ETA	81 ± 33 (34.8–160)	97 ± 60 (42.0–192)	72 ± 39 (33.6–112)	77 ± 67 (24.1–318)	87 ± 26 (41.8–118)
EA	(2.04 ± 0.71) × 10^2^ (110–375)	(2.7 ± 1.2) × 10^2^ (124–442)	(3.9 ± 1.7) × 10^2^ (203–543)	(2.6 ± 1.0) × 10^2^ (142–466)	(2.7 ± 1.0) × 10^2^ (136–414)
PEA	12 ± 8 (3.1–28.9)	23 ± 28 (1.2–71.4)	10.5 ± 6.0 (6.4–17.4)	13 ± 15 (0.1–63.4)	21 ± 18 (1.1–50.6)
MA	11 ± 8 (4.8–36.2)	26 ± 28 (4.8–73.2)	12 ± 8 (5.6–21.7)	16 ± 16 (5.1–74.3)	18 ± 11 (5.3–33.7)
SPD	39 ± 12 (25.6–68.6)	50 ± 40 (20.1–118)	37.0 ± 7.1 (29.9–44.0)	35 ± 18 (10.8–86.5)	42 ± 18 (16.6–78.3)
TYR	4.7 ± 4.0 (0.1–11.8)	6.5 ± 4.6 (0.8–12.7)	3.5 ± 2.7 (0.4–5.3)	4.4 ± 4.5 (0.5–19.8)	6 ± 8 (0.2–27.7)
HTA	28 ± 16 (6.8–59.4)	22 ± 14 (11.6–45.9)	24 ± 10 (13.6–33.2)	18 ± 13 (4.9–51.9)	31 ± 15 (8.9–56.0)

(*) Abbreviations: DMA, dimethylamine; DOP, dopamine; EA, ethylamine; ETA, ethanolamine; HTA, serotonin; MA, methylamine; PEA, phenylethylamine; SPD, spermidine; TYR, tyramine. Values in scientific notation are expressed as (mean ± SD) × 10^n^ according to ISO GUM [43].

**Table 3:** Distribution of dark chocolate samples (*n* = 91) based on origin, variety, certification, and cocoa content.

Category	Sub-Category	*n*	Cocoa Content (%)
Bean origin (Continent)	Africa	20	60–99
	South America	48	56–100
	Central America	15	66–99
	Asia and Oceania	8	70–90
Variety	Criollo	5	65–100
	Forastero	3	60–82
	Trinitario	13	70–95
	Nacional	18	56–100
	Blend	9	70–100
	Not declared	43	64–100
Certification	Organic	23	66–100
	Fairtrade	19	56–100
	Single origin	29	60–100
	Not declared	20	60–90
Cocoa content (%)	≤60	4	56–60
	61–70	32	61–70
	71–80	28	71–80
	81–90	16	81–90
	>90	11	91–100

Note: Values correspond to the information declared on product labels. The cocoa content (%) column indicates the range within each sub-category. “Not declared” indicates that the genetic variety or certification seal was not available or not reported on the product label.

## Data Availability

Data is available upon request to the authors.

## Supplementary Materials

The following supporting information can be downloaded at https://www.mdpi.com/article/10.3390/molecules31152597/s1; Figure S1. Mean concentrations (± SD) of amino acids and amines according to cocoa variety. Different lowercase letters indicate statistically significant differences according to Tukey’s HSD test (*p* < 0.05). Abbreviations: Ala, alanine; Asp, aspartic acid; Glu, glutamic acid; Cys, cysteine; Cit, citrulline; Phe, phenylalanine; Gly, glycine; Gln, glutamine; Hyp, hydroxyproline; His, histidine; Ile, isoleucine; Leu, leucine; Lys, lysine; Orn, ornithine; Pro, proline; Ser, serine; Tyr, tyrosine; Trn, threonine; Trp, tryptophan; Val, valine. DMA, dimethylamine; DOP, dopamine; EA, ethylamine; ETA, ethanolamine; HTA, serotonin; MA, methylamine; PEA, phenylethylamine; SPD, spermidine; TYR, tyramine. Figure S2. Principal component analysis (PCA) score plots of 91 commercial dark chocolate samples based on amino acid and amine profiles. (a) PCA score plot obtained from the raw data matrix. (b) PCA score plot after data correction. Samples are represented by triangles, whereas QC samples are shown as circles. Symbols are colored according to injection order. Figure S3. Distribution of cross-validated balanced classification accuracies obtained from 1000 per- mutation tests. The red vertical line indicates the performance of the optimized PLS-DA model (75.2%). None of the permuted models achieved an equal or higher classification accuracy (*p* < 0.001). Figure S4. Contribution variables plot of chocolate samples on LV2 (a) and LV3 (b). Colors indicate the brand with the highest median value for each compound. Abbreviations: Lys, lysine; EA, ethyl- amine; Cit, citrulline; PEA, phenylethylamine; Ser, serine; Tyr, tyrosine; SPD, spermidine; Leu, leu- cine; MA, methylamine; Gly, glycine; Pro, proline; HTA, hydroxytryptamine (Serotonin); Met, me- thionine; Ala, alanine; Phe, phenylalanine; Glu, glutamic acid; Cys, cysteine; Gln, glutamine; Trn, threonine; Val, valine; Ile, isoleucine; Trp, tryptophan; His, histidine. Table S1. List of analytical standards employed in the study, including compound name, abbreviation, and supplier. Table S2. Characteristics of the 91 commercial dark chocolate samples included in this study, in- cluding brand, cocoa content, declared cocoa-bean origin, cocoa variety, certification or quality seal, manufacturing location, and best-before date. Table S3. LC-MS/MS multiple reaction monitoring (MRM) acquisition parameters used for the de- termination of amino acids and amines. The table includes the analytical function of each compound (target analyte, internal standard, or surrogate standard), the number of dansyl groups after deri- vatization, precursor ion (Q1), product ion (Q3), declustering potential (DP), collision energy (CE), and collision cell exit potential (CXP). MRM transitions and instrumental parameters were adapted from the previously established method described by Navarro-Abril et al. [53]. Table S4. Verification of analytical performance for representative analytes. Repeatability was evaluated as the relative standard deviation (RSD, *n* = 3), and linearity was verified over the indicated concentration ranges. These representative compounds were selected to cover different amino acid and amine classes.

## Abbreviations

The following abbreviations are used in this manuscript:

Ala	Alanine
Asp	Aspartic acid
Asn	Asparagine
BA	Biogenic amine
BER	Balanced error rate
BUT	Butylamine
CAD	Cadaverine
Cit	Citrulline
Cys	Cysteine
DMA	Dimethylamine
DNSCl	Dansyl chloride
DOP	Dopamine
EA	Ethylamine
ETA	Ethanolamine
Gln	Glutamine
Glu	Glutamic acid
Gly	Glycine
HEX	Hexylamine
His	Histidine
HTA	5-Hydroxytryptamine (Serotonin)
Hyp	Hydroxyproline
ILE	Isoleucine
IPA	Isopropylamine
IS	Internal standard
LC-MS/MS	Liquid chromatography–tandem mass spectrometry
Leu	Leucine
LV	Latent variable
Lys	Lysine
MA	Methylamine
NQC	Nearest quality control normalization
OCT	Octopamine
Orn	Ornithine
PCA	Principal component analysis
PEA	Phenylethylamine
Phe	Phenylalanine
PLS-DA	Partial least squares-discriminant analysis
Pro	Proline
PUT	Putrescine
QC	Quality control sample
QCT	Pooled quality control sample
QMR	Repeated quality monitoring sample
SPD	Spermidine
SPM	Spermine
TA	Tyramine
Trn	Threonine
Trp	Tryptophan
Tyr	Tyrosine
Val	Valine
urep	Uncertainty due to precision

## References

[1] Food and Agriculture Organization of the United Nations “Cocoa Bean Production—UN FAO” [Dataset]. Food and Agriculture Organization of the United Nations, “Production: Crops and Livestock Products” [Original Data]. https://archive.ourworldindata.org/20260304-094028/grapher/cocoa-bean-production.html.

[2] Kongor J.E., Owusu M., Oduro-Yeboah C. (2024). Cocoa Production in the 2020s: Challenges and Solutions. CABI Agric. Biosci..

[3] Sarıtaş S., Duman H., Pekdemir B., Rocha J.M., Oz F., Karav S. (2024). Functional Chocolate: Exploring Advances in Production and Health Benefits. Int. J. Food Sci. Technol..

[4] Tan T.Y.C., Lim X.Y., Yeo J.H.H., Lee S.W.H., Lai N.M. (2021). The Health Effects of Chocolate and Cocoa: A Systematic Review. Nutrients.

[5] Braga S.C.G.N., Oliveira L.F., Hashimoto J.C., Gama M.R., Efraim P., Poppi R.J., Augusto F. (2018). Study of Volatile Profile in Cocoa Nibs, Cocoa Liquor and Chocolate on Production Process Using GC × GC-QMS. Microchem. J..

[6] Castillejos-Mijangos L.A., Meza-Márquez O.G., Osorio-Revilla G., Jiménez-Martínez C., Gallardo-Velázquez T. (2023). Identification of Variety and Prediction of Chemical Composition in Cocoa Beans (*Theobroma cacao* L.) by FT-MIR Spectroscopy and Chemometrics. Foods.

[7] Cortez D., Flores M., Calampa L.L., Oliva-Cruz M., Goñas M., Meléndez-Mori J.B., Chavez S.G. (2024). From the Seed to the Cocoa Liquor: Traceability of Bioactive Compounds during the Postharvest Process of Cocoa in Amazonas-Peru. Microchem. J..

[8] Parada T., Pardo P., Saurina J., Sentellas S. (2024). Characterization of Dark Chocolates Based on Polyphenolic Profiles and Antioxidant Activity. J. Food Sci..

[9] Sentellas S., Saurina J. (2023). Authentication of Cocoa Products Based on Profiling and Fingerprinting Approaches: Assessment of Geographical, Varietal, Agricultural and Processing Features. Foods.

[10] Calvo A.M., Botina B.L., García M.C., Cardona W.A., Montenegro A.C., Criollo J. (2021). Dynamics of Cocoa Fermentation and Its Effect on Quality. Sci. Rep..

[11] do Carmo Brito B.d.N., Campos Chisté R., da Silva Pena R., Abreu Gloria M.B., Santos Lopes A. (2017). Bioactive Amines and Phenolic Compounds in Cocoa Beans Are Affected by Fermentation. Food Chem..

[12] Oracz J., Żyżelewicz D., Nebesny E. (2015). The Content of Polyphenolic Compounds in Cocoa Beans (*Theobroma cacao* L.), Depending on Variety, Growing Region, and Processing Operations: A Review. Crit. Rev. Food Sci. Nutr..

[13] Rodriguez-Campos J., Escalona-Buendía H.B., Contreras-Ramos S.M., Orozco-Avila I., Jaramillo-Flores E., Lugo-Cervantes E. (2012). Effect of Fermentation Time and Drying Temperature on Volatile Compounds in Cocoa. Food Chem..

[14] Ullrich L., Casty B., André A., Hühn T., Chetschik I., Steinhaus M. (2023). Influence of the Cocoa Bean Variety on the Flavor Compound Composition of Dark Chocolates. ACS Food Sci. Technol..

[15] Delgado-Ospina J., Di Mattia C.D., Paparella A., Mastrocola D., Martuscelli M., Chaves-Lopez C. (2020). Effect of Fermentation, Drying and Roasting on Biogenic Amines and Other Biocompounds in Colombian Criollo Cocoa Beans and Shells. Foods.

[16] Aprotosoaie A.C., Luca S.V., Miron A. (2016). Flavor Chemistry of Cocoa and Cocoa Products—An Overview. Compr. Rev. Food Sci. Food Saf..

[17] Barišić V., Kopjar M., Jozinović A., Flanjak I., Ačkar Đ., Miličević B., Šubarić D., Jokić S., Babić J. (2019). The Chemistry behind Chocolate Production. Molecules.

[18] Kandasamy S., Yoo J., Yun J., Kang H.B., Seol K.H., Ham J.S. (2021). Quantitative Analysis of Biogenic Amines in Different Cheese Varieties Obtained from the Korean Domestic and Retail Markets. Metabolites.

[19] Zhu H., Yang S., Zhang Y., Fang G., Wang S. (2016). Simultaneous Detection of Fifteen Biogenic Amines in Animal Derived Products by HPLC-FLD with Solid-Phase Extraction after Derivatization with Dansyl Chloride. Anal. Methods.

[20] Płotka-Wasylka J., Simeonov V., Morrison C., Namieśnik J. (2018). Impact of Selected Parameters of the Fermentation Process of Wine and Wine Itself on the Biogenic Amines Content: Evaluation by Application of Chemometric Tools. Microchem. J..

[21] Spizzirri U.G., Puoci F., Iemma F., Restuccia D. (2019). Biogenic Amines Profile and Concentration in Commercial Milks for Infants and Young Children. Food Addit. Contam. Part Chem. Anal. Control Expo. Risk Assess..

[22] Balcázar-Zumaeta C.R., Fernández-Romero E., Lopes A.S., Ferreira N.R., Chagas-Júnior G.C.A., Yoplac I., López-Trigoso H.A., Tuesta-Occ M.L., Maldonado-Ramirez I., Maicelo-Quintana J.L. (2024). Amino Acid Profile Behavior during the Fermentation of Criollo Cocoa Beans. Food Chem. X.

[23] Sari A.B.T., Fahrurrozi, Marwati T., Djaafar T.F., Hatmi R.U., Purwaningsih, Wanita Y.P., Lisdiyanti P., Perwitasari U., Juanssilfero A.B. (2023). Chemical Composition and Sensory Profiles of Fermented Cocoa Beans Obtained from Various Regions of Indonesia. Int. J. Food Sci..

[24] Silveira P.T.d.S., Glória M.B.A., Tonin I.P., Martins M.O.P., Efraim P. (2023). Varietal Influence on the Formation of Bioactive Amines during the Processing of Fermented Cocoa with Different Pulp Contents. Foods.

[25] Restuccia D., Gianfranco Spizzirri U., De Luca M., Ilaria Parisi O., Picci N., Editors A., Ruggieri A., Petros Sebhatu S., Foltynowicz Z. (2016). Biogenic Amines as Quality Marker in Organic and Fair-Trade Cocoa-Based Products. Sustainability.

[26] Restuccia D., Spizzirri U.G., Puoci F., Picci N. (2015). Determination of Biogenic Amine Profiles in Conventional and Organic Cocoa-Based Products. Food Addit. Contam. Part Chem. Anal. Control Expo. Risk Assess..

[27] Dala-Paula B.M., Deus V.L., Tavano O.L., Gloria M.B.A. (2021). In Vitro Bioaccessibility of Amino Acids and Bioactive Amines in 70% Cocoa Dark Chocolate: What You Eat and What You Get. Food Chem..

[28] Pastore P., Favaro G., Badocco D., Tapparo A., Cavalli S., Saccani G. (2005). Determination of Biogenic Amines in Chocolate by Ion Chromatographic Separation and Pulsed Integrated Amperometric Detection with Implemented Wave-Form at Au Disposable Electrode. J. Chromatogr. A.

[29] Deus V.L., Resende L.M., Bispo E.S., Franca A.S., Gloria M.B.A. (2021). FTIR and PLS-Regression in the Evaluation of Bioactive Amines, Total Phenolic Compounds and Antioxidant Potential of Dark Chocolates. Food Chem..

[30] Deus V.L., Bispo E.S., Franca A.S., Gloria M.B.A. (2021). Understanding Amino Acids and Bioactive Amines Changes during On-Farm Cocoa Fermentation. J. Food Compos. Anal..

[31] Banicod R.J.S., Ntege W., Njiru M.N., Abubakar W.H., Kanthenga H.T., Javaid A., Khan F. (2025). Production and Transformation of Biogenic Amines in Different Food Products by the Metabolic Activity of the Lactic Acid Bacteria. Int. J. Food Microbiol..

[32] Özogul Y., Özogul F. (2019). Biogenic Amines Formation, Toxicity, Regulations in Food. Biogenic Amines in Food: Analysis, Occurrence and Toxicity.

[33] Ekici K., Omer A.K. (2020). Biogenic Amines Formation and Their Importance in Fermented Foods. BIO Web Conf..

[34] Omidiran A.T., Jenfa M.D. (2023). Occurrence of Biogenic Amines in Fermented Foods. Indigenous Fermented Foods for the Tropics.

[35] Wójcik W., Łukasiewicz M., Puppel K. (2021). Biogenic Amines: Formation, Action and Toxicity—A Review. J. Sci. Food Agric..

[36] Moniente M., Botello-Morte L., García-Gonzalo D., Pagán R., Ontañón I. (2022). Analytical Strategies for the Determination of Biogenic Amines in Dairy Products. Compr. Rev. Food Sci. Food Saf..

[37] Parada T., Saurina J., Sentellas S. (2025). High-Quality Dark Chocolates Identified by Liquid Chromatography-Mass Spectrometry Fingerprinting. Microchem. J..

[38] Aigensberger M., Bueschl C., Metzler-Zebeli B.U., Berthiller F., Schwartz-Zimmermann H.E. (2025). QuantyFey: An Open-Source Tool for Targeted LC-MS Quantification with Integrated Drift Correction. Anal. Chim. Acta.

[39] Thonusin C., IglayReger H.B., Soni T., Rothberg A.E., Burant C.F., Evans C.R. (2017). Evaluation of Intensity Drift Correction Strategies Using MetaboDrift, a Normalization Tool for Multi-Batch Metabolomics Data. J. Chromatogr. A.

[40] Jiang F., Liu Q., Li Q., Zhang S., Qu X., Zhu J., Zhong G., Huang M. (2020). Signal Drift in Liquid Chromatography Tandem Mass Spectrometry and Its Internal Standard Calibration Strategy for Quantitative Analysis. Anal. Chem..

[41] Kamleh M.A., Ebbels T.M.D., Spagou K., Masson P., Want E.J. (2012). Optimizing the Use of Quality Control Samples for Signal Drift Correction in Large-Scale Urine Metabolic Profiling Studies. Anal. Chem..

[42] (2024). Reference Materials—Approaches for Characterization and Assessment of Homogeneity and Stability.

[43] Joint Committee for Guides in Metrology (JCGM) (2008). Evaluation of Measurement Data—Guide to the Expression of Uncertainty in Measurement.

[44] Rojas M., Hommes A., Heeres H.J., Chejne F. (2022). Physicochemical Phenomena in the Roasting of Cocoa (*Theobroma cacao* L.). Food Eng. Rev..

[45] Silva G.S., Dala-Paula B.M., Bispo E.S., Gloria M.B.A. (2023). Bioaccessibility of Bioactive Amines in Dark Chocolates Made with Different Proportions of Under-Fermented and Fermented Cocoa Beans. Food Chem..

[46] Esposito L., Perillo M., Di Mattia C.D., Scroccarello A., Della Pelle F., Compagnone D., Sacchetti G., Mastrocola D., Martuscelli M. (2024). A Survey on Potentially Beneficial and Hazardous Bioactive Compounds in Cocoa Powder Samples Sourced from the European Market. Foods.

[47] Benes E., Matejka G., Fodor M. (2025). Near-Infrared Spectroscopy for Comprehensive Analysis of Dark Chocolate Composition. Food Chem..

[48] Afoakwa E.O., Paterson A., Fowler M., Ryan A. (2008). Flavor Formation and Character in Cocoa and Chocolate: A Critical Review. Crit. Rev. Food Sci. Nutr..

[49] Peña-Correa R.F., Mogol B.A., Fryganas C., Fogliano V. (2023). Fluidized-Bed-Roasted Cocoa Has Different Chemical Characteristics than Conventionally Roasted Cocoa. J. Agric. Food Chem..

[50] Oracz J., Nebesny E. (2014). Influence of Roasting Conditions on the Biogenic Amine Content in Cocoa Beans of Different Theobroma Cacao Cultivars. Food Res. Int..

[51] Ballabio D., Grisoni F., Todeschini R. (2018). Multivariate Comparison of Classification Performance Measures. Chemom. Intell. Lab. Syst..

[52] Jiménez-Carvelo A.M., González-Casado A., Bagur-González M.G., Cuadros-Rodríguez L. (2019). Alternative Data Mining/Machine Learning Methods for the Analytical Evaluation of Food Quality and Authenticity—A Review. Food Res. Int..

[53] Navarro-Abril A., Saurina J., Sentellas S. (2022). Simultaneous Determination of Amino Acids and Biogenic Amines by Liquid Chromatography Coupled to Mass Spectrometry for Assessing Wine Quality. Beverages.

[54] Mir-Cerdà A., Izquierdo-Llopart A., Saurina J., Sentellas S. (2021). Oenological Processes and Product Qualities in the Elaboration of Sparkling Wines Determine the Biogenic Amine Content. Fermentation.

[55] Posit Team (2025). RStudio: Integrated Development Environment for R.

[56] Kassambara A., Mundt F. (2026). Extract and Visualize the Results of Multivariate Data Analyses, R Package Factoextra Version 2.0.0.

[57] Rohart F., Gautier B., Singh A., Lê Cao K.A. (2017). mixOmics: An R Package for ‘omics Feature Selection and Multiple Data Integration. PLoS Comput. Biol..

