# Supplementary material for: Signal-Corrected LC–MS/MS Approaches in Amino Acid and Biogenic Amine Profiling for Chemometric Characterization of Commercial Dark Chocolates According to Cocoa Content and Manufacturer
Source: Molecules. 2026 Jul 25;31(15):2597. doi: 10.3390/molecules31152597 (PMC13467624; doi:10.3390/molecules31152597)
Supplement: Supplementary file 1 [file molecules-31-02597-s001.zip › molecules-4422030-supplementary.pdf]

molecules

MDPI

*Supplementary material*

# Signal-Corrected LC–MS/MS Approaches in Amino Acid and Biogenic Amine profiling for Chemometric Characterization of Commercial Dark Chocolates According to Cocoa Content and Manufacturer

**Laura V. Morales[1,2], Sonia Sentellas[1,2,3], Javier Saurina[1,2*]**

[1] Department of Chemical Engineering and Analytical Chemistry, Universitat de Barcelona, Martí I Franquès 1-11, 08028, Barcelona, Spain.

[2] Institut de Recerca en Nutrició i Seguretat Alimentària, Universitat de Barcelona, 08921 Santa Coloma de Gramenet, Spain.

[3] Serra Húnter Fellow Programme, Generalitat de Catalunya, Via Laietana 2, 08003 Barcelona, Spain.

* Correspondence: JS: xavi.saurina@ub.edu; LVM: lmoralese@ub.edu

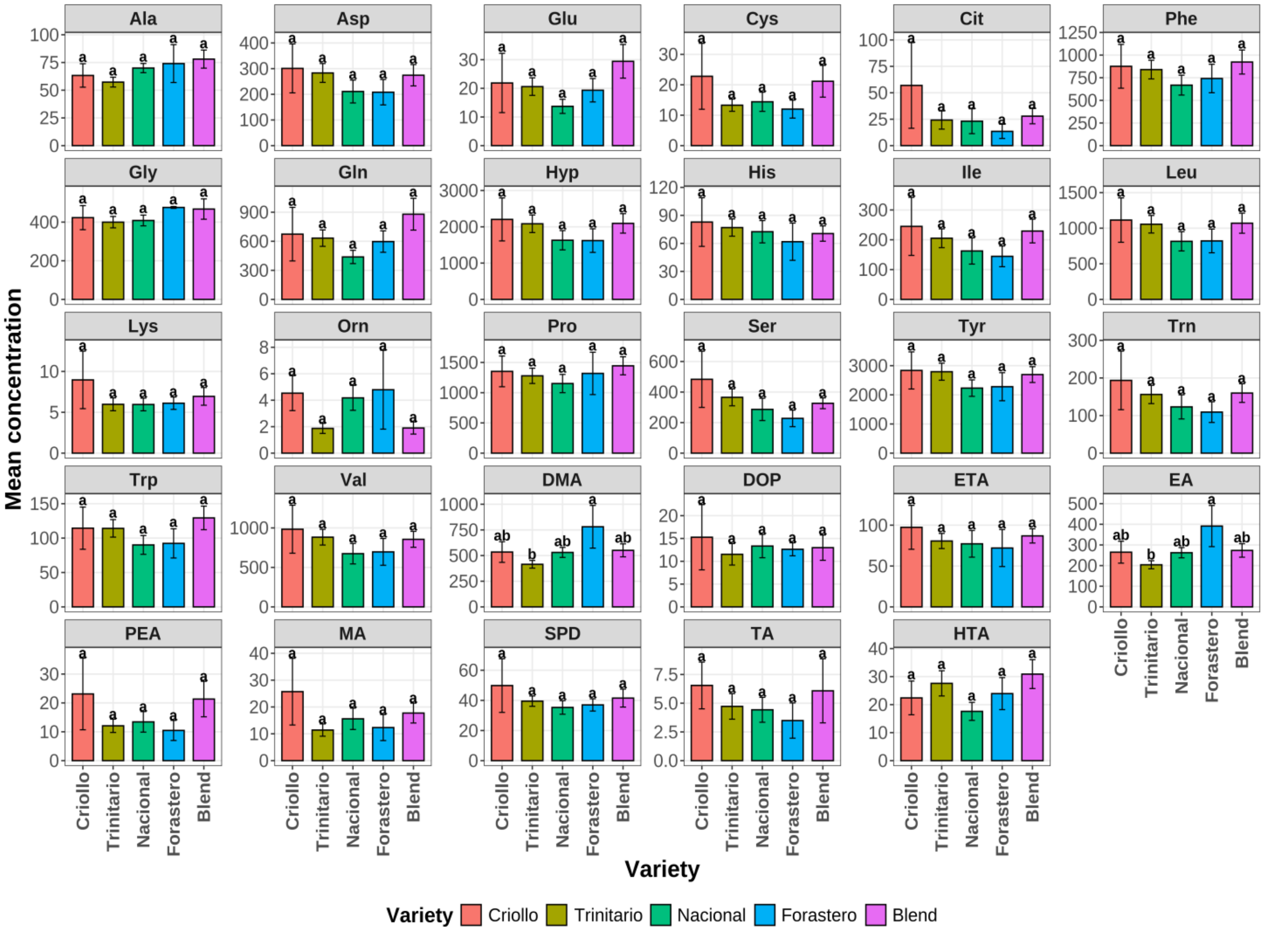


**Figure S1.** Mean concentrations (± SD) of amino acids and amines according to cocoa variety. Different lowercase letters indicate statistically significant differences according to Tukey's HSD test ($p < 0.05$). Abbreviations: Ala, alanine; Asp, aspartic acid; Glu, glutamic acid; Cys, cysteine; Cit, citrulline; Phe, phenylalanine; Gly, glycine; Gln, glutamine; Hyp, hydroxyproline; His, histidine; Ile, iso-

leucine; Leu, leucine; Lys, lysine; Orn, ornithine; Pro, proline; Ser, serine; Tyr, tyrosine; Trn, threonine; Trp, tryptophan; Val, valine. DMA, dimethylamine; DOP, dopamine; EA, ethylamine; ETA, ethanolamine; HTA, serotonin; MA, methylamine; PEA, phenylethylamine; SPD, spermidine; TYR, tyramine.

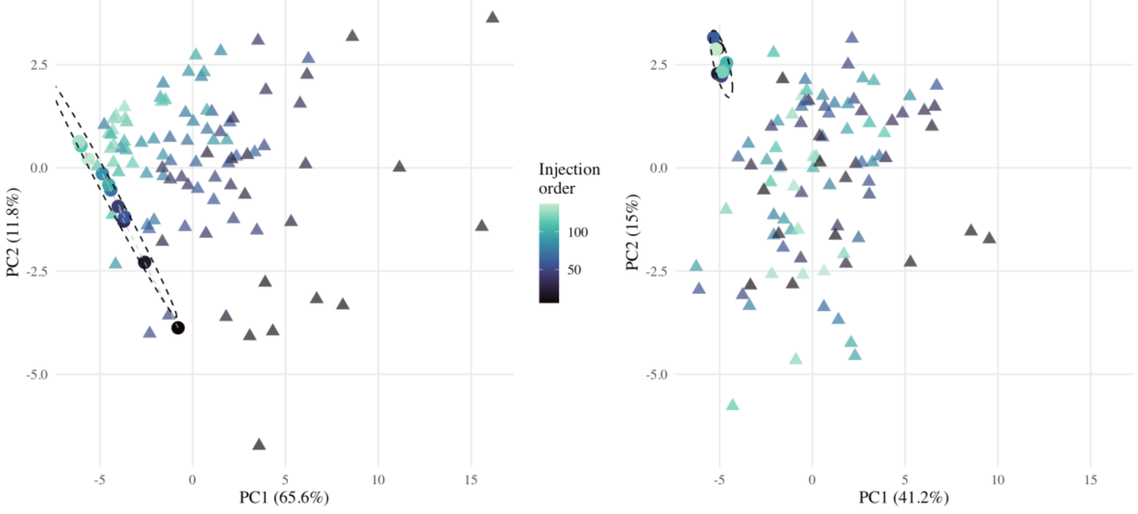


**(a)** **(b)**

**Figure S2.** Principal component analysis (PCA) score plots of 91 commercial dark chocolate samples based on amino acid and amine profiles. (a) PCA score plot obtained from the raw data matrix. (b) PCA score plot after data correction. Samples are represented by triangles, whereas QC samples are shown as circles. Symbols are colored according to injection order.

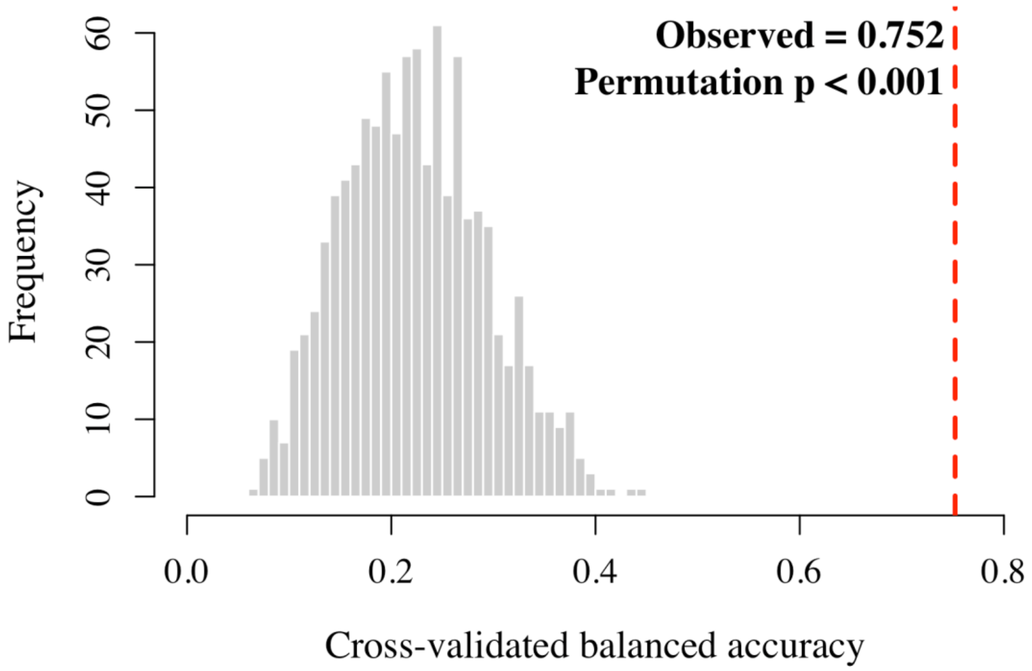


**Figure S3.** Distribution of cross-validated balanced classification accuracies obtained from 1000 permutation tests. The red vertical line indicates the performance of the optimized PLS-DA model (75.2%). None of the permuted models achieved an equal or higher classification accuracy ($p < 0.001$).

**Table S1.** List of analytical standards employed in the study, including compound name, abbreviation, and supplier.

| Compound | Abbreviation | Supplier |
|---|---|---|
| Hydroxyproline | Hyp | Fluka (Buchs, Switzerland) |
| proline | Pro | |
| L-glutamic acid | Glu | |
| L-aspartic acid | Asp | |
| L-lysine monohydrochloride | Lys | |
| 2-phenylethylamine hydrochloride | PEA | |
| L-tyrosine | Tyr | Sigma Aldrich (Steinheim, Germany) |
| L-cysteine | Cys | |
| Dopamine hydrochloride | DOP | |
| L-leucine | Leu | |
| Serotonin hydrochloride | HTA | |
| L-glutamine | Gln | |
| L-ornithine monohydrochloride | Orn | Carlo Erba (Milan, Italy) |
| L-serine | Ser | |
| L-asparagine | Asn | |
| Putrescine dihydrochloride | PUT | Alfa Aesar (Massachusetts, USA) |
| Cadaverine dihydrochloride | CAD | |
| Dimethylamine hydrochloride | DMA | |
| Spermine tetrahydrochloride | SPM | |
| Spermidine trihydrochloride | SPD | |

| L-Citrulline | Cit | Chemika (St. Gallen, Switzerland) |
| --- | --- | --- |
| Ethylamine hydrochloride | EA | Tokyo Chemical Industry (TCI, Tokyo, Japan) |
| Ethanolamine hydrochloride | ETA | |
| Methylamine hydrochloride | MA | |
| Tyramine hydrochloride | TYR | |
| Hexylamine hydrochloride | HEX | |
| Butylamine hydrochloride | BUT | |
| Isopropyl amine hydrochloride | IPA | |
| Octopamine | OCT | |
| L-histidine | His | Thermo Scientific (Waltham, Massachusetts, USA) |
| L-tryptophan | Trp | |
| L-isoleucine | Ile | |
| L-valine | Val | |
| L-glycine | Gly | Merck (Darmstadt, Germany) |
| L-methionine | Met | |
| L-alanine | Ala | |
| Threonine | Trn | |
| L-phenylalanine | Phe | |

**Table S2.** Characteristics of the 91 commercial dark chocolate samples included in this study, including brand, cocoa content, declared cocoa-bean origin, cocoa variety, certification or quality seal, manufacturing location, and best-before date.

| Code | Brand | Cocoa content (%) | Bean origin (Country (Continent)) | Variety | Certification/Seal | Location of manufacture | Best before date |
| --- | --- | --- | --- | --- | --- | --- | --- |
| X01 | Succhard | 70 | Dominican Republic (CA) | Trinitario | Organic | Madrid, Spain | 2025 |
| X02 | Blanxart | 80 | Ghana (Af) | Not declared | Fairtrade | Barcelona, Spain | 2025 |
| X03 | Torras | 100 | Unspecified (SA) | Blend | Organic | Girona, Spain | 2025 |
| X04 | Chocolate Amatller | 85 | Ghana (Af) | Not declared | Single origin | Barcelona, Spain | 2026 |
| X05 | Blanxart | 72 | Dominican Republic (CA) | Not declared | Organic | Barcelona, Spain | 2026 |
| X06 | Blanxart | 99 | Dominican Republic (CA) | Not declared | Organic | Barcelona, Spain | 2026 |
| X07 | Chocolate Amatller | 85 | Ecuador (SA) | Not declared | Single origin | Barcelona, Spain | 2026 |
| X08 | Ethiquable | 98 | Ecuador (SA) | Nacional | Fairtrade | Italy | 2025 |
| X09 | Ethiquable | 85 | Madagascar (Af) | Not declared | Fairtrade | Italy | 2026 |
| X10 | Ethiquable | 72 | Haiti (CA) | Not declared | Fairtrade | Italy | 2027 |
| X11 | Republica del cacao | 75 | Ecuador (SA) | Nacional | Single origin | Quito, Ecuador | 2025 |
| X12 | Republica del cacao | 75 | Ecuador (SA) | Nacional | Single origin | Quito, Ecuador | 2025 |
| X13 | Millesime | 74 | Guatemala (CA) | Trinitario | Fairtrade | Lliege, Belgium | 2025 |

| | | | | | | | |
|---|---|---|---|---|---|---|---|
| X14 | Blanxart | 72 | Ghana (Af) | Not declared | Fairtrade | Barcelona, Spain | 2025 |
| X15 | Succhard | 85 | Dominican Republic (CA) | Trinitario | Organic | Madrid, Spain | 2025 |
| X16 | JD Gross | 95 | Ecuador (SA) | Nacional | Fairtrade | Murcia, Spain | 2025 |
| X17 | JD Gross | 85 | Ecuador (SA) | Nacional | Fairtrade | Murcia, Spain | 2027 |
| X18 | Museu Xocolata | 70 | Ghana (Af) | Forastero | Not declared | Barcelona, Spain | 2025 |
| X19 | Museu Xocolata | 70 | Madagascar (Af) | Trinitario | Single origin | Barcelona, Spain | 2025 |
| X20 | Anvers Fortuna | 100 | Peru (SA) | Nacional | Single origin | Tasmania, Australia | 2025 |
| X21 | Museu Xocolata | 70 | Tanzania (Af) | Trinitario | Single origin | Barcelona, Spain | 2025 |
| X22 | Museu Xocolata | 70 | Dominican Republic (CA) | Trinitario | Organic | Barcelona, Spain | 2025 |
| X23 | Xocolata Jolonch | 72 | Venezuela (SA) | Not declared | Not declared | Lleida, Spain | 2025 |
| X24 | Blanxart | 99 | Ghana (Af) | Not declared | Fairtrade | Barcelona, Spain | 2026 |
| X25 | Blanxart | 81 | Peru (SA) | Not declared | Organic | Barcelona, Spain | 2025 |
| X26 | Blanxart | 91 | Congo (Af) | Not declared | Single origin | Barcelona, Spain | 2026 |
| X27 | Blanxart | 78 | Brazil (SA) | Not declared | Organic | Barcelona, Spain | 2025 |
| X28 | Blanxart | 87 | Nicaragua (CA) | Not declared | Organic | Barcelona, Spain | 2025 |
| X29 | Blanxart | 95 | Ecuador (SA) | Trinitario | Single origin | Barcelona, Spain | 2025 |
| X30 | Aruntam | 72 | India (A&O) | Not declared | Organic | Milan, Italy | 2025 |
| X31 | Chocolat Bonnat | 75 | Sri Lanka (A&O) | Not declared | Not declared | Cedex, France | 2025 |
| X32 | Kaitxo | 70 | Philippines (A&O) | Blend | Not declared | Bizkaia, Spain | 2025 |
| X33 | Utopick | 90 | Philippines (A&O) | Not declared | Not declared | Valencia, Spain | 2025 |
| X34 | Utopick | 70 | Philippines (A&O) | Not declared | Not declared | Valencia, Spain | 2026 |
| X35 | Chef Kankel | 75 | Philippines (A&O) | Trinitario | Not declared | Abelda de Iregua, Spain | 2025 |
| X36 | Blanxart | 82 | Dominican Republic (CA) | Not declared | Fairtrade | Barcelona, Spain | 2025 |
| X37 | Blanxart | 100 | Peru (SA) | Criollo | Organic | Barcelona, Spain | 2025 |
| X38 | Blanxart | 93 | Congo (Af) | Not declared | Single origin | Barcelona, Spain | 2026 |
| X39 | Blanxart | 77 | Peru (SA) | Criollo | Single origin | Barcelona, Spain | 2026 |
| X40 | Cacao hunters | 64 | Colombia (SA) | Not declared | Not declared | Popayan, Colombia | 2025 |
| X41 | Blanxart | 82 | Congo (Af) | Forastero | Single origin | Barcelona, Spain | 2025 |
| X42 | Blanxart | 85 | Nicaragua (CA) | Not declared | Organic | Barcelona, Spain | 2026 |

| | | | | | | | |
|---|---|---|---|---|---|---|---|
| X43 | Xocolata Jolonch | 70 | New guinea (A&O) | Not declared | Not declared | Lleida, Spain | 2025 |
| X44 | Xocolata Jolonch | 66 | Madagascar (Af) | Not declared | Not declared | Lleida, Spain | 2025 |
| X45 | Xocolata Jolonch | 66 | Mexico (CA) | Not declared | Not declared | Lleida, Spain | 2025 |
| X46 | Sprüngli | 65 | Venezuela (SA) | Criollo | Not declared | Zurich, Switzerland | 2025 |
| X47 | Sprüngli | 75 | Bolivia (SA) | Criollo | Not declared | Zurich, Switzerland | 2025 |
| X48 | Sprüngli | 70 | Cuba (CA) | Trinitari o | Not declared | Zurich, Switzerland | 2025 |
| X49 | Sprüngli | 60 | Ghana (Af) | Foraster o | Not declared | Zurich, Switzerland | 2025 |
| X50 | Republica del cacao | 75 | Ecuador (SA) | Nacional | Single origin | Quito, Ecuador | 2025 |
| X51 | Succhard | 70 | Dominican Republic (CA) | Trinitari o | Fairtrade | Madrid, Spain | 2025 |
| X52 | JD Gross | 56 | Unspecified (SA) | Nacional | Fairtrade | Murcia, Spain | 2026 |
| X53 | Kallari and Yumbos | 85 | Ecuador (SA) | Nacional | Organic | Quito, Ecuador | 2025 |
| X54 | Sancao | 75 | Colombia (SA) | Blend | Not declared | Medellin, Colombia | 2025 |
| X55 | Paccari | 72 | Ecuador (SA) | Nacional | Organic | Quito, Ecuador | 2025 |
| X56 | Dorigenn | 70 | Colombia (SA) | Nacional | Fairtrade | Tumaco, Colombia | 2026 |
| X57 | Mindus | 77 | Ecuador (SA) | Nacional | Organic | Quito, Ecuador | 2025 |
| X58 | Kallari and Yumbos | 75 | Ecuador (SA) | Not declared | Organic | Quito, Ecuador | 2025 |
| X59 | Kallari and Yumbos | 70 | Ecuador (SA) | Nacional | Single origin | Quito, Ecuador | 2025 |
| X60 | Kallari and Yumbos | 60 | Ecuador (SA) | Nacional | Single origin | Quito, Ecuador | 2025 |
| X61 | Chocolate Amatller | 72 | Nicaragua (CA) | Blend | Organic | Barcelona, Spain | 2026 |
| X62 | Chocolate Amatller | 83 | Peru (SA) | Blend | Organic | Barcelona, Spain | 2026 |
| X63 | Blanxart | 76 | Brazil (SA) | Trinitari o | Single origin | Barcelona, Spain | 2026 |
| X64 | JD Gross | 70 | Ecuador (SA) | Nacional | Fairtrade | Murcia, Spain | 2026 |
| X65 | Xocolata Jolonch | 72 | Peru (SA) | Not declared | Not declared | Lleida, Spain | 2026 |
| X66 | Xocolata Jolonch | 80 | Colombia (SA) | Not declared | Not declared | Lleida, Spain | 2025 |
| X67 | Chocolate Amatller | 74 | Madagascar (Af) | Trinitari o | Organic | Barcelona, Spain | 2026 |
| X68 | Chocolate Santander | 70 | Colombia (SA) | Blend | Single origin | Medellin, Colombia | 2026 |
| X69 | Kallari and Yumbos | 85 | Ecuador (SA) | Not declared | Single origin | Quito, Ecuador | 2025 |
| X70 | Paccari | 65 | Ecuador (SA) | Nacional | Single origin | Quito, Ecuador | 2025 |
| X71 | Paccari | 70 | Ecuador (SA) | Nacional | Organic | Quito, Ecuador | 2025 |
| X72 | Republica del cacao | 75 | Ecuador (SA) | Not declared | Single origin | Quito, Ecuador | 2025 |

| | | | | | | | |
|---|---|---|---|---|---|---|---|
| X73 | Kallari and Yumbos | 70 | Ecuador (SA) | Not declared | Organic | Quito, Ecuador | 2025 |
| X74 | Paccari | 60 | Ecuador (SA) | Nacional | Single origin | Quito, Ecuador | 2025 |
| X75 | Dorigenn | 70 | Colombia (SA) | Blend | Fairtrade | Bogota, Colombia | 2025 |
| X76 | Dorigenn | 70 | Colombia (SA) | Blend | Fairtrade | Bogota, Colombia | 2025 |
| X77 | Tierra madre | 70 | São Tomé and Príncipe (Af) | Not declared | Fairtrade | No reported, Spain | 2025 |
| X78 | Chocolate Amatller | 81 | Uganda (Af) | Blend | Organic | Barcelona, Spain | 2027 |
| X79 | Ethiquable | 100 | Peru (SA) | Not declared | Fairtrade | Fleurance, France | 2026 |
| X80 | Millesime | 80 | Mexico (CA) | Criollo | Fairtrade | Lliege, Belgium | 2025 |
| X81 | Chocolate Amatller | 70 | Ghana (Af) | Not declared | Single origin | Barcelona, Spain | 2025 |
| X82 | Nau Do Cacau | 66 | São Tomé and Príncipe (Af) | Not declared | Organic | Leiria, Portugal | 2025 |
| X83 | Puchero | 85 | Tanzania (Af) | Trinitari o | Single origin | Valladolid, Spain | 2025 |
| X84 | Fjak | 80 | Tanzania (Af) | Not declared | Single origin | Hardanger, Norway | 2026 |
| X85 | Moro | 75 | New guinea (A&O) | Not declared | Not declared | Llerena, Spain | 2025 |
| X86 | Tibito | 70 | Colombia (SA) | Not declared | Single origin | Bogota, Colombia | 2025 |
| X87 | Tibito | 70 | Colombia (SA) | Not declared | Single origin | Bogota, Colombia | 2025 |
| X88 | Tibito | 70 | Colombia (SA) | Not declared | Single origin | Bogota, Colombia | 2025 |
| X89 | Tibito | 70 | Colombia (SA) | Not declared | Single origin | Bogota, Colombia | 2025 |
| X90 | Tibito | 70 | Colombia (SA) | Not declared | Single origin | Bogota, Colombia | 2025 |
| X91 | Cacao hunters | 70 | Colombia (SA) | Not declared | Not declared | Popayan, Colombia | 2026 |

CA, Central America and the Caribbean; SA, South America; Af, Africa; A&O, Asia and Oceania. "Not declared" indicates that the information was not provided on the product label. "Blend" refers to chocolates produced from a mixture of cocoa varieties. Cocoa content corresponds to the percentage declared by the manufacturer.

**Table S3**. LC-MS/MS multiple reaction monitoring (MRM) acquisition parameters used for the determination of amino acids and amines. The table includes the analytical function of each compound (target analyte, internal standard, or surrogate standard), the number of dansyl groups after derivatization, precursor ion (Q1), product ion (Q3), declustering potential (DP), collision energy (CE), and collision cell exit potential (CXP). MRM transitions and instrumental parameters were adapted from the previously established method described by Navarro-Abril et al. [53].

| Compound | Abbreviation | Analytical function | Number of dansyl groups | Q1 (m/z) | Q3 (m/z) | DP (V) | CE (V) | CXP (V) |
|---|---|---|---|---|---|---|---|---|
| L-Alanine | Ala | Target | 1 | 323 | 170 | 100 | 50 | 12 |
| L-Aspartic acid | Asp | Target | 1 | 367 | 170 | 100 | 50 | 12 |
| L-Citrulline | Cit | Target | 1 | 409 | 170 | 100 | 50 | 12 |
| L-Cysteine | Cys | Target | 1 | 355 | 170 | 90 | 30 | 10 |
| L-Glutamic acid | Glu | Target | 1 | 381 | 170 | 100 | 50 | 12 |
| L-Glutamine | Gln | Target | 1 | 380 | 170 | 100 | 50 | 12 |
| Glycine | Gly | Target | 1 | 309 | 170 | 100 | 50 | 12 |
| L-Histidine | His | Target | 2 | 622 | 170 | 100 | 50 | 12 |
| Hydroxyproline | Hyp | Target | 1 | 365 | 170 | 100 | 50 | 12 |
| L-Isoleucine | Ile | Target | 1 | 365 | 170 | 100 | 50 | 12 |
| L-Leucine | Leu | Target | 1 | 365 | 170 | 100 | 50 | 12 |
| L-Lysine monohydrochloride | Lys | Target | 2 | 613 | 170 | 100 | 50 | 12 |
| L-Methionine | Met | Target | 1 | 383 | 170 | 100 | 50 | 12 |
| L-Ornithine monohydrochloride | Orn | Target | 2 | 599 | 170 | 100 | 50 | 12 |
| L-Phenylalanine | Phe | Target | 1 | 399 | 170 | 100 | 50 | 12 |
| L-Proline | Pro | Target | 1 | 349 | 170 | 100 | 50 | 12 |
| L-Serine | Ser | Target | 1 | 339 | 170 | 100 | 50 | 12 |
| Threonine | Trn | Target | 1 | 353 | 170 | 100 | 50 | 12 |
| L-Tryptophan | Trp | Target | 1 | 438 | 170 | 100 | 50 | 12 |
| L-Tyrosine | Tyr | Target | 2 | 648 | 170 | 100 | 50 | 12 |
| L-Valine | Val | Target | 1 | 351 | 170 | 100 | 50 | 12 |
| Cadaverine dihydrochloride | CAD | Target | 2 | 569 | 234 | 100 | 50 | 12 |
| Dimethylamine hydrochloride | DMA | Target | 1 | 279 | 170 | 100 | 50 | 12 |
| Dopamine hydrochloride | DOP | Target | 1 | 387 | 170 | 100 | 50 | 12 |
| Ethanolamine hydrochloride | ETA | Target | 1 | 295 | 170 | 100 | 50 | 12 |
| Ethylamine hydrochloride | EA | Target | 1 | 279 | 170 | 100 | 50 | 12 |
| Methylamine hydrochloride | MA | Target | 1 | 265 | 170 | 100 | 50 | 12 |
| 2-Phenylethylamine hydrochloride | PEA | Target | 1 | 355 | 170 | 90 | 30 | 10 |

| **Compound** | **Abbreviation** | **Analytical function** | **Number of dansyl groups** | **Q1 (m/z)** | **Q3 (m/z)** | **DP (V)** | **CE (V)** | **CXP (V)** |
|---|---|---|---|---|---|---|---|---|
| Putrescine dihydrochloride | PUT | Target | 2 | 555 | 234 | 120 | 60 | 12 |
| Serotonin hydrochloride | HTA | Target | 1 | 410 | 170 | 100 | 50 | 8 |
| Spermidine trihydrochloride | SPD | Target | 3 | 845 | 360 | 40 | 80 | 12 |
| Spermine tetrahydrochloride | SPM | Target | 4 | 1135 | 360 | 90 | 50 | 12 |
| Tyramine hydrochloride | TYR | Target | 1 | 604 | 234 | 100 | 50 | 12 |
| L-Asparagine | Asn | Internal standard | 1 | 366 | 170 | 100 | 50 | 12 |
| Isopropylamine hydrochloride | IPA | Internal standard | 1 | 293 | 170 | 100 | 50 | 12 |
| Octopamine | OCT | Internal standard | 1 | 620 | 170 | 66 | 59 | 12 |
| Butylamine hydrochloride | BUT | Surrogate standard | 1 | 307 | 170 | 125 | 50 | 14 |
| Hexylamine hydrochloride | HEX | Surrogate standard | 1 | 335 | 157 | 86 | 39 | 12 |

**Table S4.** Verification of analytical performance for representative analytes. Repeatability was evaluated as the relative standard deviation (RSD, $n$ = 3), and linearity was verified over the indicated concentration ranges. These representative compounds were selected to cover different amino acid and amine classes.

| Compound | Abbreviation | Repeatability (RSD, %, $n$ = 3) | Linear range (mg kg$^{-1}$) | $R^2$ |
|---|---|---|---|---|
| Alanine | Ala | 3.5 | 0.1–5 | 0.991 |
| Aspartic acid | Asp | 5 | 0.1–35 | 0.986 |
| Citrulline | Cit | 6.4 | 0.2–25 | 0.995 |
| Cysteine | Cys | 6.8 | 0.05–10 | 0.998 |
| Glutamic acid | Glu | 6.6 | 0.1–35 | 0.992 |
| Glycine | Gly | 7.1 | 0.5–20 | 0.989 |
| Histidine | His | 7.9 | 0.5–7 | 0.997 |
| Hydroxyproline | Hyp | 7.3 | 1–35 | 0.996 |
| Isoleucine | Ile | 4.4 | 0.5–35 | 0.991 |
| Leucine | Leu | 4.7 | 0.5–27 | 0.995 |
| Ornithine | Orn | 8.6 | 0.1–5 | 0.989 |
| Phenylalanine | Phe | 5.3 | 0.2–25 | 0.995 |
| Proline | Pro | 7 | 0.8–36 | 0.996 |
| Serine | Ser | 8.7 | 0.5–25 | 0.986 |
| Tryptophan | Trp | 4.4 | 0.1–10 | 0.997 |
| Tyrosine | Tyr | 4.7 | 0.8–35 | 0.992 |
| Valine | Val | 4.2 | 0.2–30 | 0.996 |
| Dopamine | DOP | 7.7 | 0.1–20 | 0.985 |
| Ethanolamine | ETA | 6.1 | 0.8–30 | 0.987 |
| Ethylamine | EA | 2.3 | 0.6–30 | 0.984 |
| Phenylethylamine | PEA | 4.6 | 0.1–10 | 0.999 |

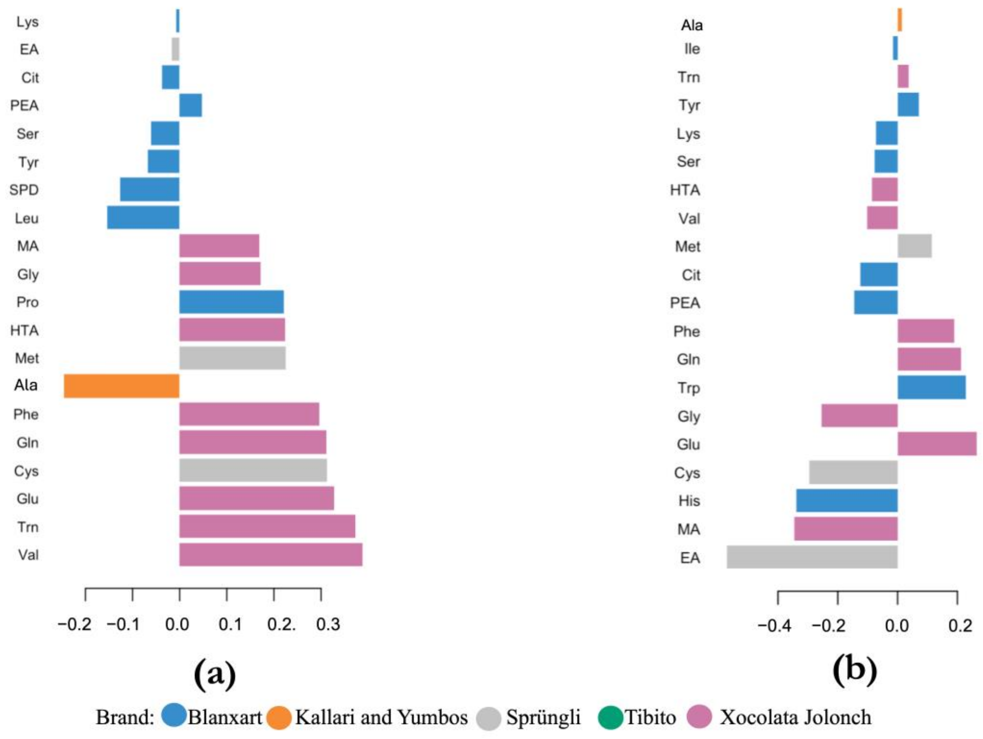


**Figure S4.** Contribution variables plot of chocolate samples on LV2 (a) and LV3 (b). Colors indicate the brand with the highest median value for each compound. Abbreviations: Lys, lysine; EA, ethylamine; Cit, citrulline; PEA, phenylethylamine; Ser, serine; Tyr, tyrosine; SPD, spermidine; Leu, leucine; MA, methylamine; Gly, glycine; Pro, proline; HTA, hydroxytryptamine (Serotonin); Met, methionine; Ala, alanine; Phe, phenylalanine; Glu, glutamic acid; Cys, cysteine; Gln, glutamine; Trn, threonine; Val, valine; Ile, isoleucine; Trp, tryptophan; His, histidine.
